# ﻿A revision of the millipede family Paracortinidae (Diplopoda, Callipodida)

**DOI:** 10.3897/zookeys.1187.113473

**Published:** 2023-12-28

**Authors:** Nesrine Akkari, Oliver Macek, Pavel Stoev

**Affiliations:** 1 Naturhistorisches Museum Wien, Burgring 7, 1010 Wien, Austria Naturhistorisches Museum Wien Wien Austria; 2 National Museum of Natural History at the Bulgarian Academy of Sciences, Tsar Osvoboditel Blvd. 1, 1000 Sofia, Bulgaria National Museum of Natural History at the Bulgarian Academy of Sciences Sofia Bulgaria; 3 Pensoft Publishers, Prof. G. Zlatarski Str. 12, Sofia, Bulgaria Pensoft Publishers Sofia Bulgaria

**Keywords:** China, descriptions, gonopods, identification key, new genus, new species, taxonomy, Vietnam

## Abstract

The taxonomy of the family Paracortinidae Wang & Zhang, 1993 is revised based on literature, old and recently collected material. A new genus *Crassipetalum* Akkari & Stoev, **gen. nov.** is described, to accommodate a new species *Crassipetalummagnum* Akkari & Stoev, **gen. nov. et sp. nov.** and a recently described species *Crassipetaluminflatum* (Chen, Zheng & Jian, 2023), **comb. nov.** The genus *Scotopetalum* Shear, 2000 hitherto described for the Vietnamese species *S.warreni* Shear, 2000 and subsequently synonymised with the genus *Paracortina* Wang & Zhang, 1993 is here resurrected and supplemented with another species, *S.chinensis* (Stoev & Geoffroy, 2004), **comb. nov.**, ex *Paracortinachinensis* Stoev & Geoffroy, 2004. The status of the fourth genus in the family, *Angulifemur* Zhang, 1997, is reconfirmed. Based on recently collected specimens from China, two new species of the genus *Paracortina* are described: *P.asciformis* Akkari & Stoev, **sp. nov.** (Sichuan Prov., Lixian County) and *P.kabaki* Akkari & Stoev, **sp. nov.** (Yunnan, Shangrila County). The Vietnamese species *Paracortinamultisegmentata* Stoev & Geoffroy, 2004 and *Paracortinakyrang* Nguyen, Stoev, Nguyen & Vu, 2023 are considered of uncertain taxonomic position within Paracortinidae. Differential diagnoses for the proposed genera as well as for all the species are presented, and descriptions or descriptive notes for all the species are provided, using a standardised terminology, and when possible, accompanied by micrographs of the habitus and gonopod structures. An identification key based on gonopod structures is proposed for all the members of the family. A discussion on species affinities, secondary sexual characters, troglomorphic characters, geographical distribution, and habitat preferences are also provided together with a distribution map for all members of the family.

## ﻿Introduction

The South-East Asian family Paracortinidae Wang & Zhang, 1993 is known to comprise two genera, viz., *Paracortina* Wang & Zhang, 1993 (13 species) and *Angulifemur* Zhang, 1997 (two species). All species occur in China, except *Paracortinawarreni* (Shear, 2000), *P.multisegmentata* Stoev & Geoffroy, 2004, and *P.kyrang* Nguyen, Stoev, Nguyen & Vu, 2023, which are known from north Vietnam (Shear 2000; Stoev and Geoffroy 2004; Enghoff et al. 2015; Nguyen et al. 2023). Despite being the subject of a number of studies (Wang and Zhang 1993; Shear 2000; Stoev 2004; Stoev and Geoffroy 2004; Stoev et al. 2008; Enghoff et al. 2015; Liu and Tian 2015; Chen et al. 2023; Nguyen et al. 2023), the taxonomy of this family and its genera is still far from being settled. The original diagnosis of the family was based on a combination of a few habitual and gonopod characters, namely “the presence of 2–5 setae on pleurotergites 1–4, 6–7 setae on pleurotergite 5, all in the back of segments 6 to penultimate, females with reduced leg-pair 2 and divided cyphopods, males with gonopods presenting a large cone-shaped sternal process, and two large prefemoral processes” (Wang and Zhang 1993: 386). The family was originally described to enclose three genera, *Paracortina* Wang & Zhang, 1993, *Altum* Wang & Zhang, 1993, and *Relictus* Wang & Zhang, 1993, of which, latter two were later downgraded to a subgeneric rank by Wang (1996) (see also Wang and Mauriès 1996).

The preliminary “phylogenetic analysis” of the family (Wang 1996) is quite controversial and not in accordance with the taxonomy of the family. Shear (2000) questioned the validity of the family and described a new monospecific genus from Vietnam, namely *Scotopetalum* Shear, 2000, which is morphologically similar to the other paracortinids but that he assigned to the European-Asia Minor family Schizopetalidae. Shear (2000) commented on the variability of the setal distribution in Callipodida and discussed the origin of the processes as interpreted by Wang and Zhang (1993). The same author attempted to homologise the gonopodal structures, pointing to the similarities between the Schizopetalidae and Paracortinidae and criticising the use of setal pattern as a character to separate the different genera.

Stoev (2004), Stoev and Geoffroy (2004), and later Enghoff et al. (2015) presented a list of external characters that in their opinion could justify the validity of the family Paracortinidae. These are pleurotergal crests well developed, poriferous ones prominent; head in males convex; coxal pores on leg-pairs 3–23; gonosternum reduced or fused with coxae; gonocoxae freely connected through a medial membranous lamina; telopodite with one or two prefemoroidal processes, their apical parts usually covered with macrosetae; basal and proximal parts of femoroidal stem simple, unbranched; distal part usually broadened, with several processes; a parasolenomere always present; leg 2 in females reduced to two simple sclerites. Stoev (2004) described *Paracortinawangi* Stoev, 2004, which he later synonymised with *Angulifemurunidigitis* Zhang, 1997 (Stoev et al. 2008), considering a potential synonymy of *Angulifemur* with *Paracortina* (see also Liu and Tian 2015). However, in the latest taxonomic treatment of the family (Enghoff et al. 2015) *Angulifemur* and *Paracortina* are kept as separate genera. Stoev and Geoffroy (2004) synonymised the (sub)genera *Altum*, *Relictus*, and *Scotopetalum* with *Paracortina*, and refined the diagnosis of the family, presenting an identification key to all paracortinid species, based on external morphology and gonopod structures.

Recently, Liu and Tian (2015) described two morphologically similar species of genus *Paracortina* from caves in Guizhou and Guangxi provinces of China, viz., *P.zhangi* Liu & Tian, 2015 and *P.yinae* Liu & Tian, 2015. Nguyen et al. (2023) described *Paracortinakyrang* Nguyen, Stoev, Nguyen & Vu, 2023 from a cave in northern Vietnam, and Chen et al. (2023) described *Paracortinainflata* Chen, Zheng & Jiang, 2023 from Yintiaoling in China.

In addition to Paracortinidae, two other extant callipodidan families occur in Southeast Asia, namely the Caspiopetalidae Lohmander, 1931, which is mostly distributed in Central Asia (one genus, eight species), with a single outlying species *Bollmaniaberoni* Stoev & Enghoff, 2005 from a cave in Yunnan, China (Stoev and Enghoff 2005) and Sinocallipodidae Zhang, 1993 (one genus, six species), the latter being the only family of Callipodida restricted to the tropics (Stoev and Enghoff 2011).

In this work, we redefine and revise the taxonomy of the family Paracortinidae, describing one new species of a new genus *Crassipetalum* gen. nov., and two new species of the genus *Paracortina*. Moreover, we resurrect the genus *Scotopetalum* to accommodate *Scotopetalumwarreni* and *S.chinensis* (Stoev & Geoffroy 2004), comb. nov. Due to their highly derived gonopod morphology, we question the exact position of the species *P.multisegmentata* and *P.kyrang* in the genus *Paracortina*.

Descriptive notes using a standard terminology for the gonopod structures and micrographs are provided for representatives of all genera and most species to help understanding the complex gonopod configuration. The family does in our understanding include 19 species in four genera that we delimit based on a number of apomorphies in the gonopod structures. We provide the most relevant citations for the species as an exhaustive list. The repositories of the respective types were provided in earlier publications (Stoev et al. 2008; Enghoff et al. 2015; Liu and Tian 2015; Chen et al. 2023; Nguyen et al. 2023).

A discussion addressing the taxonomic affinities of the different species is presented along with a review of the different secondary sexual characters and their potential importance in delimiting taxa. Additional notes on the troglomorphic features and the geographic distribution of the species are provided together with a distribution map for all members of the family.

## ﻿Materials and methods

The material was obtained from different museums (see list of repositories), studied and photographed using a Nikon DS-Ri2 camera mounted on a Nikon SMZ25 stereomicroscope, using NIS-Elements Microscope Imaging Software with an Extended Depth of Focus (EDF). Obtained images were edited in Adobe Photoshop CS6 and assembled in Adobe InDesign CS6. The map is performed used QGIS 3.28.9 (Firenze) with Open Topography-DEM-Downloader 2.0 plugin and WGS 84.

Acronyms of the repositories:

**IZCAS**National Zoological Museum of China, Institute of Zoology, Chinese Academy of Sciences, Beijing, China.

**NMNHS**National Museum of Natural History, Bulgarian Academy of Sciences, Sofia, Bulgaria.

**ZMUM** Zoological Museum of Moscow University, Moscow, Russia.

List of abbreviations:

**a** lobe-like mesal process of coxa

**b** (sub-)falcate mesal process of coxa

**c** coxa

**Ca** Coxal anterior lobe

**Cl** Coxal lateral lobe

**dl** distal lamella of telopodite

**dp** distal process of telopodite

**k** lateral process of the distal part of telopodite

**M** mesal process of the distal part of telopodite

**n** notch on the distal part of the telopodite

**p** lateral process of the proximal part of telopodite

**pf1** prefemoroidal process 1

**pf2** prefemoroidal process 2

**pr** prefemur

**ps** parasolenomere

**PT** pleurotergites

**s** solenomere

**T** telopodite

**tb** blunt tooth of telopodite

**Tp** telopodital projection

**tr** trochanter

## ﻿Taxonomic account

### ﻿Order Callipodida Pocock, 1894

#### ﻿Family Paracortinidae

Taxon classificationAnimaliaCallipodidaParacortinidae

Wang & Zhang, 1993

DDF155A8-95C3-5A43-BE78-2B7108430802

##### Emended diagnosis.

Middle-sized callipodidans with well-developed pleurotergal crests, poriferous ones prominent; male head either unmodified or with a prominent bulge. Pleurotergal setae apically pointed, usually in anterior position until PT4, on PT 5 some setae migrate posteriorly, and from PT6 all are in posterior position. Gonopods: parallel, diverging or converging. Sternum reduced or fused with coxae; coxae freely connected through a medial membranous lamina. Each gonopod with one or two prefemoroidal processes clavate and setose (**pf1, pf2**); one or two coxal lobes and a mesal coxal process varying in size; telopodite (**T**) long, unbranched in proximal parts, sometimes curved, twisted or forming a sharp angle at mid-length, distally complex with apical folds and lamellae and smaller projections, ending with solenomere (**s**) and parasolenomere (**ps**). Leg 2 in adult females reduced to two simple sclerites.

In most representatives of the family we examined, the chaetotaxy in the anterior pleurotergites is the same and follows this distribution (Table 1), except in the Vietnamese species *Paracortinamultisegmentata* and *P.kyrang*. As already established by Wang and Zhang (1993), some paracortinids have a greater number of setae on each hemipleurite (6 or 7) from PT 6 onwards. However, the majority of the species show a 5+5 pattern.

**Table 1.** Chaetotaxy of anterior pleurotergites in species of the family Paracortinidae, except in the Vietnamese species *P.multisegmentata* Stoev & Geoffroy, 2004 and *P.kyrang* Nguyen, Stoev, Nguyen & Vu, 2023.

	Anterior setae	Posterior setae
Collum	*a*, *b*, *c*, *d*, *e*	–
PT 2	*a*, *b*, *c*, *d*, *e*	–
PT 3	*a*, *b*, *c*, *d*, *e*	–
PT 4	*a*, *b*, *c*, *d*, *e*	–
PT 5	*a*, *d*	*b*, *c*, *e*
PT 6	–	*a*, *b*, *c*, *d*, *e*

##### Included genera.

*Angulifemur*Zhang 1997 – two species.

*Crassipetalum* Akkari & Stoev, gen. nov. – two species.

*Paracortina* Wang & Zhang, 1993 – 13 species.

*Scotopetalum*Shear 2000, stat. rev. – two species.

#### ﻿Genus Angulifemur

Taxon classificationAnimaliaCallipodidaParacortinidae

Zhang, 1997

0AD7FE6B-EA27-5F77-9BE8-1B6882300F65

##### Type species.

*Angulifemurtridigitis* Zhang, 1997.

##### Included species.

*Angulifemurtridigitis* Zhang, 1997; *Angulifemurunidigitis* Zhang, 1997.

##### Diagnosis.

Head with no projection on vertex, leg-pair 7 with a long subfalcate mesal process and a broader triangular one on coxa. Gonopods diverging from the base. Each gonopod with two short, clavate, uniformly setose prefemoroidal processes (**pf1** and **pf2**); reduced coxal processes: (**a**) an anterior lobe-like projection and (**b**) a generally reduced thin, hyaline subfalcate or cone-shaped process not reaching telopodite’s mid-length. Telopodite (**T**) with a stout stem forming an angular projection at ~ 1/3 to mid-length, distal part twisting and sharply narrowing, tapering towards its apex. Telopodite distally twisted, with one (*A.unidigitis*) or more (*A.tridigitis*) spine-like or tooth-like processes. Differs from *Paracortina* and *Scotopetalum* by the diverging stems of telopodite and from the genus *Crassipetalum* gen. nov. by the much smaller prefemoroidal process/es and a subfalcate coxal process (**b**) never surpassing the telopodite.

#### ﻿ Angulifemur tridigitis

Taxon classificationAnimaliaCallipodidaParacortinidae

Zhang, 1997

53A04282-895D-5D48-ACE3-A6D6047BB88F

Fig. 28

Angulifemur tridigitis Zhang, 1997: 2, figs 1–11.

##### Diagnosis.

It can easily be distinguished from *A.unidigitis* by having larger falcate coxal process (**b**) nearly reaching mid-length of the telopodite, much less sinuous distal part of telopodite (**T**) and more angular projection (**Tp**). Distal part of the telopodite differing in the presence of two downturned spine-like processes (vs one in *A.unidigitis*).

##### Comments.

Known only from the original description (Zhang 1997).

##### Distribution.

Known only from Yang-fen and Niupeng-yanzi caves in Mengzi County, Yunnan, China (Fig. 28)

#### ﻿ Angulifemur unidigitis

Taxon classificationAnimaliaCallipodidaParacortinidae

Zhang, 1997

0A287487-C7D7-5FC9-8005-B5E7B7CD41E8

**Figure 1.**
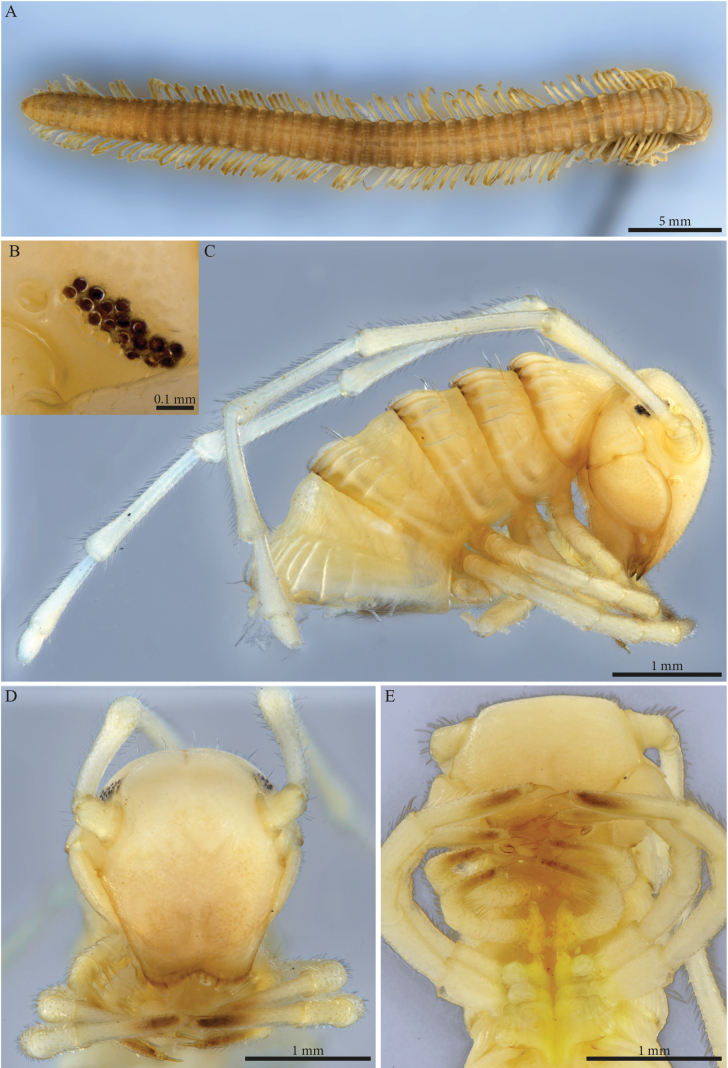
*Angulifemurunidigitis* Zhang, 1997, male (BG-NMNHS-INV-000000006260 NMNHS) **A** middle and posterior part of body, dorsal view **B** head: organ of Tömösváry and ommatidia **C–E** head and anterior pleurotergites **C** lateral view **D** frontal view **E** ventral view.

**Figure 2.**
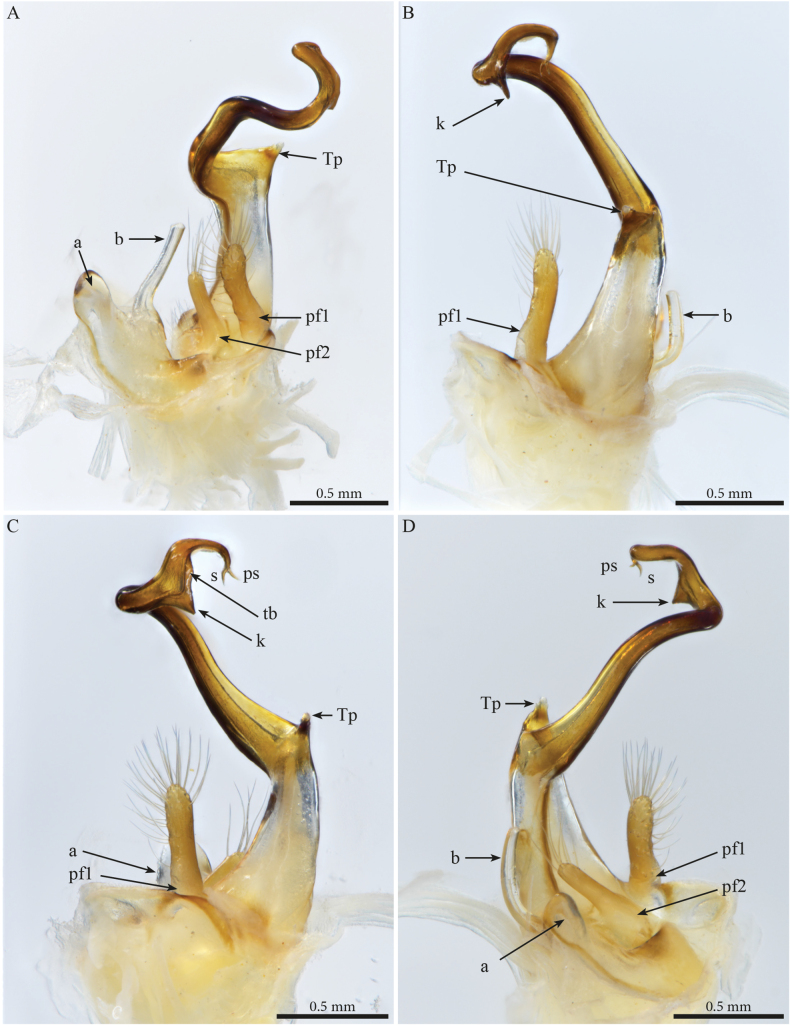
*Angulifemurunidigitis* Zhang, 1997, male (BG-NMNHS-INV-000000006260 NMNHS), right gonopod **A** anterior view **B** posterior view **C** lateral view **D** mesal view. Abbreviations: a = mesal process of coxa; b = falcate mesal process of coxa; pf1 = prefemoroidal process 1; pf2 = prefemoroidal process 2; k = lateral process of the distal part of telopodite; ps = parasolenomere; s = solenomere; tb = blunt tooth of telopodite; Tp = telopodital projection.

Figs 1 , 2 , 26A, B , 28

Angulifemur unidigitis Zhang, 1997: 2, figs 12–15.
Paracortina wangi Stoev, 2004: 2, figs 1–11; Stoev et al. 2008: 17 (synonymisation).

##### Material examined.

1 female, China, Yunnan, Menzi County, Wulichong Sinkhole Cave (No 3), 04.01.1989, P. Beron leg. (BG-NMNHS-INV-000000006259 NMNHS), Stoev det. 25.07.2003, 1 male, China, Yunnan, Menzi County, Long Bao Pao Dong (cave), 07.01.1989, P. Beron leg. (BG-NMNHS-INV-000000006260 NMNHS).

##### Diagnosis.

It can easily be distinguished from *A.tridigitis* by the much shorter and upright coxal process, a distally more sinuous telopodite, bearing a pointed process at its mid length (**Tp**); distal part of telopodite with one tooth-shaped process (**k**).

##### Descriptive notes.

Habitus matching the description of *Paracortinawangi* (Stoev 2004). The species is characterised by long walking legs and antennae, absence of projection on head vertex in adult males, reduced number of ommatidia and a strongly enlarged organ of Tömösváry (Fig. 1).

##### Male sexual characters.

Leg-pairs 1 and 2 reduced and more setose than the rest, showing prefemoral and tarsal brushes (Fig. 1E), leg-pair 2 with a small anterior process and posterior gonopore, leg-pair 3 with a smaller triangular process on coxa (Fig. 1E), leg-pair 6 with one small hyaline triangular mesal process on coxa, prefemur showing proximally a constriction on the posterior margin (Fig. 26A), leg-pair 7 with two coxal processes (Fig. 26B), a long subfalcate mesal process and a shorter, larger, subtriangular one (corresponding to *f* and *t*, respectively in Stoev 2004: fig. 6), coxal sacs (Fig. 1E) present on leg-pair 3–23.

***Gonopods*** (Fig. 2). Each gonopod with two short, clavate, setose prefemoroidal processes (**pf1** and **pf2**), with **pf1** slightly larger and more setose than **pf2** (Fig. 2A, C). Coxa with two reduced processes on the median margin: (**a**) a rounded lobe directed mesad (Fig. 2A, C, D), and (**b**) a reduced, cone-shaped one, pointing distad (Fig. 2 A, B, D). Telopodital (**T**) stem proximally broad, with a pointed triangular projection (**Tp**) at the third to mid-length of its posterior margin, marking an abrupt twist and narrowing of the process (Fig. 2A–D). Distal half of **T** sinusoidal, gently narrowing distad, and bent laterad. Distal part of **T** darkly pigmented, with a sharp triangular tooth (**k**) and a much smaller blunt one (**tb**) on the lateral margin (Fig. 2B–D). **T** further narrowing distad, marking a complete loop, its apex projecting in a short curved downturned process, bifurcated into solenomere (**s**) and parasolenomere (**ps**).

##### Comments.

The specimens studied here are part of the type series of the species Paracortina (Altum) wangi Stoev, 2004. Stoev (2004) described this millipede as a new species of the genus *Paracortina* and subgenus Altum based on the presence of five posterior setae on the sixth hemipleurite. While describing *P.wangi*Stoev (2004) was unaware of the publication of Zhang (1997). Being also described from the same type locality (Longbaopo-Wulichong cave system) in Mengzi County, Stoev et al. (2008) subsequently synonymised *P.wangi* with *Angulifemurunidigitis*.

##### Distribution.

Until now, the species is known only from Long Bao Pao - Wulichong cave system and Laoxiao Cave, Mengzi County, China (Fig. 28).

#### ﻿ Crassipetalum

Taxon classificationAnimaliaCallipodidaParacortinidae

Akkari & Stoev gen. nov.

7A27E232-99A7-507E-9E68-8FD7A36F2ACC

https://zoobank.org/07C4E7B1-8D0C-407D-B932-F564546568A7

##### Type species.

*Crassipetalummagnum* Akkari & Stoev, sp. nov.

##### Included species.

*Crassipetalummagnum* Akkari & Stoev, sp. nov.; *Crassipetaluminflatum* (Chen, Zheng & Jian, 2023), comb. nov.

##### Diagnosis.

Head with no projection on vertex, leg-pairs 6 and 7 without noticeable modifications. Gonopods parallel, distal part of the telopodites crossing. Each gonopod with a large clavate prefemoroidal process (**pf1**) reaching the distal part of telopodite (and sometimes accompanied by a smaller one); coxa with a protruding rounded anterior projection and a large coxal process, latter almost of the same size as the telopodite. Telopodite (**T**) with a stout stem, distally expanding in three main folds including a horizontal mesal projection accommodating the solenomere (**s**) and parasolenomere (**ps**). Different from all the other genera of Paracortinidae by the enlarged mesal falcate coxal process and prefemoroidal process, and by the shape of the distal part of the telopodites.

##### Etymology.

A combination of *crassus*, meaning fat/stout, referring to the large mesal coxal process and *petalum* a suffix used in many genera in the order Callipodida.

#### ﻿ Crassipetalum magnum

Taxon classificationAnimaliaCallipodidaParacortinidae

Akkari & Stoev sp. nov.

1382C116-E4ED-58BA-A2D8-2E467C812F56

https://zoobank.org/B9A36E4F-DAAF-4FD5-9670-551A38D3B62A

Figs 3 , 4 , 28

##### Material examined.

***Holotype***: adult male, China, Gansu Province, Cha-gang Village, Zhou-qu County, alt. 1650 m., on 12.05.1998, leg. Chen De-niu, Zhang Guo-qia (TM_206979 IZCAS).

##### Etymology.

Species epithet refers to the unusually large mesal coxal and prefemoroidal processes.

##### Diagnosis.

Different from *Crassipetaluminflatum* by the larger and elongated shape of the prefemoroidal process, the absence of a second prefemoroidal process, and the different distal part of telopodite.

##### Description.

**Figure 3.**
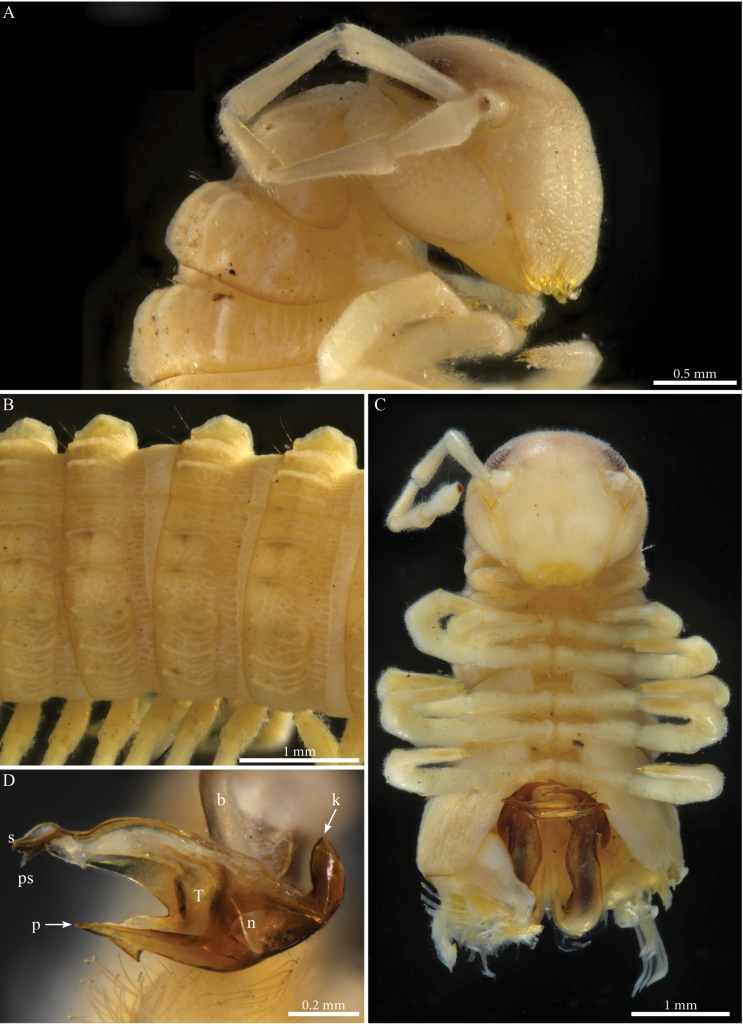
*Crassipetalummagnum* Akkari & Stoev, gen. nov., sp. nov., male holotype (No. TM_206979 IZCAS) **A** head and anterior pleurotergites, laterofrontal view **B** midbody pleurotergites, dorsolateral view **C** head and anterior pleurotergites with gonopods in situ, ventral view **D** right gonopod, distal part, anterior view. Abbreviations: b = falcate mesal process of coxa; k = lateral process of the distal part of telopodite; n = notch on the distal part of the telopodite; p = lateral process on the distal part of telopodite; ps = parasolenomere; s = solenomere; T = telopodite.

Body cylindrical, length 37–38 mm, maximal width ca 2.2 mm at PT6; body narrowing anteriad and posteriad from PT6; 54 (52 + 2 apodous) pleurotergites (PTs) + telson. Live colour unknown. Preserved specimen with yellow to pale brownish metazona (Fig. 3A, B); prozona greyish white (Fig. 3B); no stripes or other particular colour patterns; legs yellowish (Fig. 3C). Head: same colour as the body, vertex slightly darker, antennae whitish yellow (Fig. 3A). Fields of ommatidia subtriangular, blackish, composed of ~ 65 transparent ommatidia in eight rows from dorsal to ventral. Organ of Tömösváry ~ 2–3 × larger than ommatidium, situated close to and touching anterior side of eye. Head convex, with no particular modifications (Fig. 3C), covered with minute setae. Antennae short, extending backwards to around mid-length of PT5 (Fig. 3A, C); length of antennomeres (mm): 1 = 0.2; 2 = 0.87; 3 = 0.7; 4 = 0.76; 5 = 0.32; 6 = 0.45; 7 = 0.19. PTs composed of smooth prozona and carinate metazona, latter being greater in diameter than prozona. Prozona without crests, anterior part of metazona with scale-like ornamentation followed by a sharply raising posterior part forming well-developed longitudinal narrow and subparallel crests (Fig. 3B), well-separated from one another and extending over whole-body ring; crests gradually reduced in size laterally and ventrally. Chaetotaxy: all setae in anterior position on PTs 1–4, setae *b*, *c*, *e* migrated posteriorly on PT5; all setae on posterior position from PT6 onwards. Crests well developed, also on collum, comprising primary and secondary series; collum with seven or eight crests on each hemipleurite. Ozopores visible from sixth to the 49^th^PT, located on fourth (largest) crest. Hypoproct tripartite, median sclerite largest, subrectangular, bearing a pair of basal macrosetae; lateral sclerites smaller, triangular, with one seta each. Paraprocts divided into large ventral and smaller dorsal sclerites, each paraproct with a pair of long macrosetae. Spinnerets long and slender, arising from the caudal edge of epiproct and extending well beyond the margins of paraprocts.

##### Male sexual characters.

PTs 6 and 7 enlarged, leg-pairs 1 and 2 reduced and more setose than the rest, showing femoral and tarsal “brushes”, leg-pair 2 with posterior gonopore, legs 1–7 without noticeable modifications (Fig. 3C), coxal sacs present (visible) on leg-pairs 3–16.

**Figure 4.**
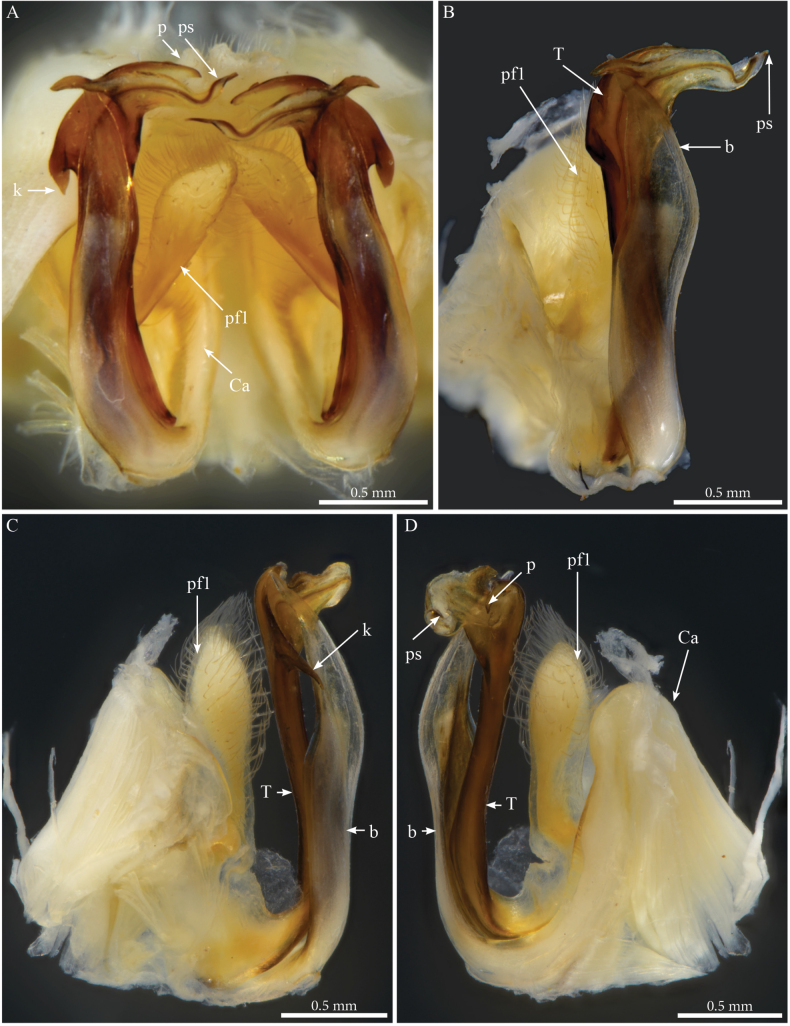
*Crassipetalummagnum* Akkari & Stoev, gen. nov., sp. nov. male holotype (No. TM_206979 IZCAS), gonopods **A** ventral view **B** right gonopod, posterolateral view **C** lateral view **D** anteromesal view. Abbreviations: b = falcate mesal process of coxa; Ca = coxal lobe; k = lateral process of the distal part of telopodite; p = lateral process of the proximal part of telopodite; ps = parasolenomere; s = solenomere; T = telopodite.

***Gonopods*** (Figs 3C, D, 4). Parallel, distally crossing. Each gonopod with one elongated, setose, clavate prefemoroidal process (**pf1**), reaching the distal part of telopodite (Fig. 4A, C, D). Setae of prefemoroidal process large and dense. Coxa with a protruding rounded anterior lobe (**Ca**) (Fig. 4D) and a large coxal process (**b**), almost of the same size as telopodite (Fig. 4), narrowing at mid-length and apically projecting in a pointed tip lodged in small lateral notch of the distal part of the telopodite (Fig. 3D). Telopodite (**T**) with a stout stem, distally with a large lateral triangular downturned projection (**k**), a large mesal notch (**n**) separating a mesal spur-like process (**p**) and a posterior plateau-like horizontal process with a bifurcated mesal projection accommodating the solenomere (**s**) and parasolenomere (**ps**).

**Female** unknown.

##### Distribution.

Zhou-qu County, Cha-gang Village, China (Fig. 28).

#### ﻿ Crassipetalum inflatum

Taxon classificationAnimaliaCallipodidaParacortinidae

(Chen, Zheng & Jian, 2023) comb. nov.

FB9EACC1-BB8E-5140-A774-627DEA27F18B

Paracortina inflata Chen, Zheng & Jian, 2023: 54–57, figs 7–10.

##### Diagnosis.

Different from *Crassipetalummagnum* Akkari & Stoev, sp. nov. by the ovoid shape of the prefemoroidal process, the presence of a second, smaller prefemoroidal process, and by the differently shaped and more complex distal part of the telopodite (Chen et al. 2023).

##### Distribution.

Hongqi Longtan Cave, Yintiaoling National Nature Reserve, Wuxi County, Chongqing, China (Fig. 28).

#### ﻿Genus Paracortina

Taxon classificationAnimaliaCallipodidaParacortinidae

Wang & Zhang, 1993

860523A1-A247-510B-A927-9D5399129202

= Altum Wang & Zhang, 1993: 381.
= Relictus Wang & Zhang, 1993: 378.

##### Type species.

*Paracortinaleptoclada* Wang & Zhang, 1993.

##### Included species.

*P.asciformis* Akkari & Stoev, sp. nov. – Lixian County, Sichuan, China.

*P.carinata* (Wang & Zhang, 1993) – Shangrila County (=Zhong Dian/ Zhongdian County), Yunnan, China.

*P.kabaki* Akkari & Stoev, sp. nov. – Shangrila County, Yunnan, China.

*P.kyrang* Nguyen, Stoev, Nguyen & Vu, 2023 – Ky Rang Cave, Quoc Toan Commune, Quang Hoa District, Cao Bang, Vietnam.

*P.leptoclada* Wang & Zhang, 1993 – Shangrila County, Yunnan, China.

*P.multisegmentata* Stoev & Geoffroy, 2004 – Ngọc Lặc County, Thanh Hoa District, Vietnam.

*P.serrata* (Wang & Zhang, 1993) – Deqin County, Yunnan, China.

*P.stimula* (Wang & Zhang, 1993) – Shangrila County, Yunnan, China.

*P.thallina* (Wang & Zhang, 1993) – Batang County, Sichuan, and Shangrila County, Yunnan, China.

*P.viriosa* (Wang & Zhang, 1993) – Shangrila County, Yunnan, and Mang kang County/Markam? County, Tibet Autonomous Region, China.

*P.voluta* Wang & Zhang, 1993 – Yajiang County and Yanyuan County (new record), Sichuan, China.

*P.yinae* Liu & Tian, 2015 – Cave in Yanchang Village, Guangxi, China.

*P.zhangi* Liu & Tian, 2015 – Cave Qiaoxia Dong, Guizhou, China.

##### Diagnosis.

The type genus of the family Paracortinidae, which differs from *Angulifemur* by having parallel stems of telopodites; from *Scotopetalum* by the presence of large anteromedian subfalcate coxal process, and from the genus *Crassipetalum* gen. nov. by the much smaller prefemoroidal process/es and a subfalcate coxal process **b** (never surpassing the telopodite).

#### ﻿ Paracortina asciformis

Taxon classificationAnimaliaCallipodidaParacortinidae

Akkari & Stoev sp. nov.

799AD075-515C-5378-8B2C-C5876454A37C

https://zoobank.org/73289E5B-1469-43C2-8573-6B3D812886AE

**Figure 5.**
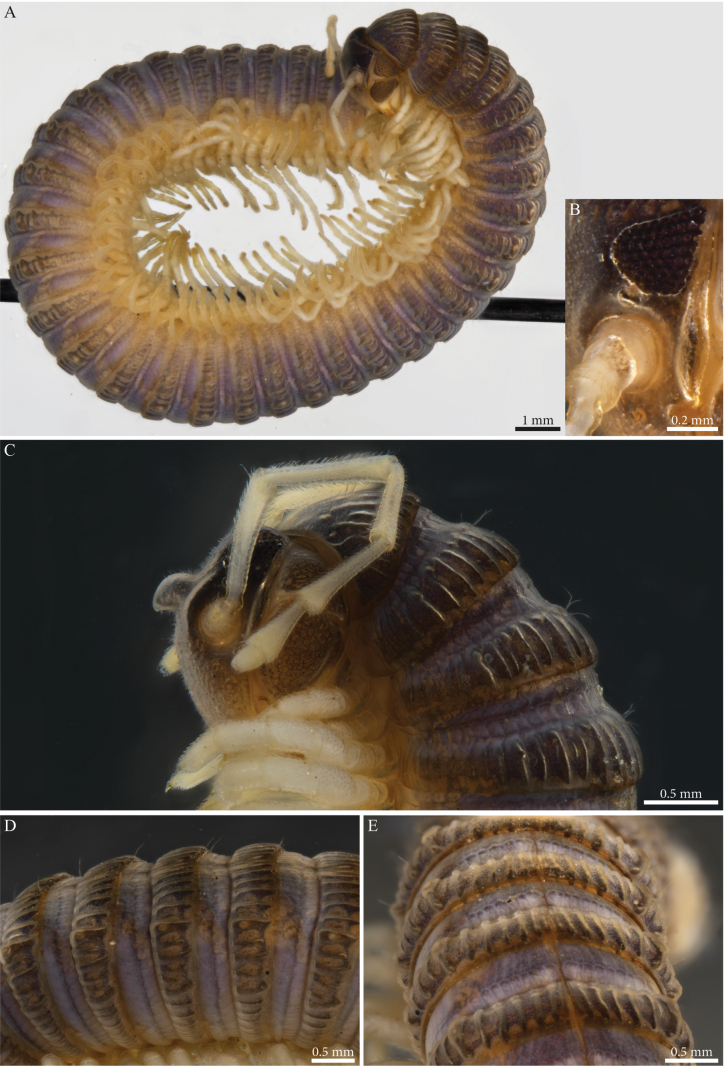
*Paracortinaasciformis* Akkari & Stoev, sp. nov. **A, B, D, E** Female paratype (Rd 5348 ZMUM) **A** anterior body lateral view **B** head left side, organ of Tömösváry, and ommatidia **C** male holotype (Rd 5347 ZMUM) head and anterior pleurotergites, lateral view **D, E** midbody pleurotergites, **D** lateral view **E** dorsal view.

**Figure 6.**
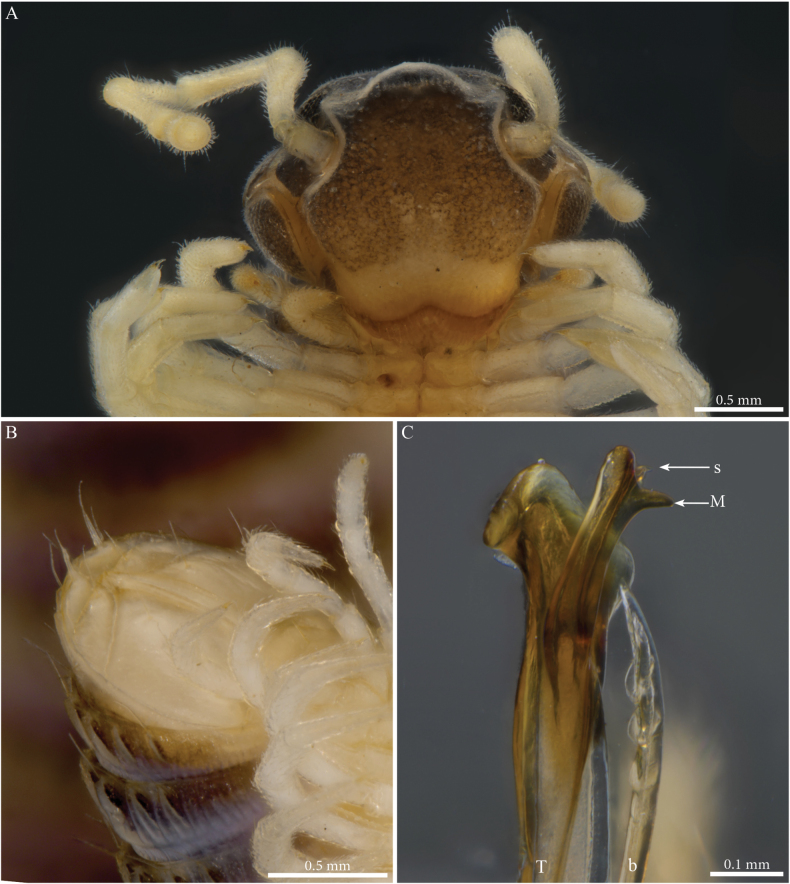
*Paracortinaasciformis* Akkari & Stoev, sp. nov., male holotype (Rd 5347 ZMUM) **A** head and anterior pleurotergites, frontal view **B** telson and posterior part of body, posterolateral view **C** right gonopod mesal coxal process and distal part of telopodite, posterior view. Abbreviations: b = falcate mesal process of coxa; M = mesal process of the distal part of telopodite; s = solenomere; T = telopodite.

**Figure 7.**
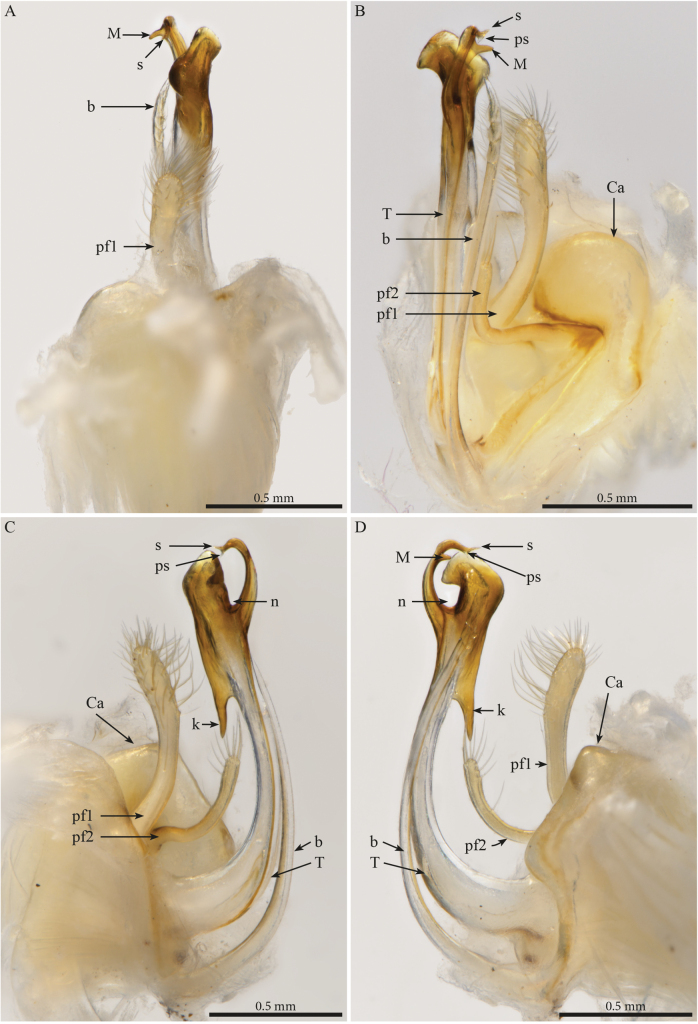
*Paracortinaasciformis* Akkari & Stoev, sp. nov., 1 male holotype (Rd 5347 ZMUM), right gonopod **A** anterior view **B** posterior view **C** lateral view **D** mesal view. Abbreviations: b = falcate mesal process of coxa; Ca = anterior lobe of coxa; pf1 = prefemoroidal process 1; pf2 = prefemoroidal process 2; k = lateral process of the distal part of telopodite; M = mesal process of the distal part of telopodite; n = notch on the distal part of the telopodite; ps = parasolenomere; s = solenomere; T = telopodite.

Figs 5 , 6 , 7 , 27E , 28

##### Material examined.

***Holotype***: 1 adult male China, Sichuan Prov., Lixian County, SW of Tonghua Village, 31°33'29"N, 103°19'36"E, 08.07.2012, alt. 1905 m, I. Belousov & G. Davidian leg. (Rd 5347 ZMUM); ***paratype***: 1 adult female, 60 PTs + telson, same data as holotype (Rd 5348 ZMUM).

##### Etymology.

The species epithet *ascia* + *formis*, referring to the distal shape of the telopodite having a shape of an axe in lateral view. Adjective.

##### Diagnosis.

Different from all other species of the genus *Paracortina* by the distinctive shape of the distal part of telopodite resembling an axe.

##### Description.

Length 39 mm, maximal width ca 2.3 mm at PT6; body narrowing anteriorly and posteriorly from PT6; 60 (59 + 1 apodous) pleurotergites + telson. Live colour unknown. Preserved specimen dark brown, metazona dorsally dark brown, especially on crests, laterally and ventrally paler; prozona greyish; legs yellowish (Figs 5A, 6A). Head: frontal part yellowish, vertex slightly dark brown, antennae yellow (Figs 5C, 6A).

Fields of ommatidia subtriangular, blackish, composed of ~ 56 transparent ommatidia in eight or nine rows (Fig. 5B). Organ of Tömösváry ~ 2 × an ommatidium situated close to and touching anterior side of eye. Antennae moderately long (Figs 5C, 6A). Length of antennomeres (mm): 1 = 0.12; 2 = 0.93; 3 = 0.86; 4 = 0.58; 5 = 0.65; 6 = 0.33; 7 = 0.11. PTs composed of smooth prozona and carinate metazona (Fig. 5D, E), latter being more pronounced and greater in diameter in the posterior part. Prozona void of crests, anterior part of metazona with low carinae followed by a sharply raising posterior part forming well-developed longitudinal narrow and subparallel crests, well-separated from one another; crests gradually reduce in size laterally and ventrally (Fig. 5C–E). Chaetotaxy follows the pattern of all setae being in anterior position on PTs 1–4, setae *b*, *c*, *e* migrating posteriorly on PT5 and all setae posteriorly on PT6 onwards. Crests moderately developed, also on collum, comprising alternating primary and secondary series, primary slightly higher than secondary; collum with ca nine crests on each hemipleurite. Ozopores visible from 6^th^ to 59^th^PT, located on 6^th^ (largest) PT. Hypoproct tripartite, median sclerite largest, subrectangular, bearing a pair of basal macrosetae; lateral sclerites smaller, triangular, with one seta each. Paraprocts divided into large ventral and smaller dorsal sclerites, each pair with a pair of long macrosetae. Spinnerets long and slender, arising from the caudal edge of epiproct and extending well beyond the margins of paraprocts (Fig. 6B).

##### Male sexual characters.

Head with a pronounced beak-shaped projection (Figs 6C, 7A), covered with minute setae. Leg-pairs 1 and 2 reduced and more setose than the rest, leg-pair 2 with a small anterior process and posterior opening of the gonopores, leg-pair 7 with a protruding curved mesal process pointing laterad and a shorter subtriangular one on coxa, trochanter with an anterior triangular projection covered with setae (Fig. 27E) Coxal sacs present (noticeable) on leg-pairs 3–13/16.

***Gonopods*.** Parallel, each gonopod with two short, slender, clavate, asymmetrical, and apically setose prefemoroidal processes **pf1** and **pf2** (Fig. 7B–D); coxa with a low rounded lobe on the anterior margin (**Ca**), one long falcate mesal coxal process (**b**), reaching the distal part of the telopodite, its distal part showing ca four beaded structures (Fig. 6C). Telopodite (**T**) stout, with a broad stem proximally, gradually narrowing until its distal third before expanding in two main darkly pigmented parts separated by a rounded notch (**n**), the larger part axe-shaped (Fig. 7C, D), showing a round apical margin and a sharp triangular opposite end (**k**), second part as a slender curved stem, apically bifurcated in solenomere (**s**) and parasolenomere (**ps**) and bearing a smaller subapical triangular tooth (**M**) (Fig. 7).

##### Distribution.

Known only from its type locality (Fig. 28).

#### ﻿ Paracortina carinata

Taxon classificationAnimaliaCallipodidaParacortinidae

(Wang & Zhang, 1993)

E3DF3BCF-FC15-537D-A932-BA2F0FBA5B92

Fig. 28

Altum carinatum Wang & Zhang, 1993: 385, figs 29–32.
Paracortina carinata : Stoev and Geoffroy 2004: 94.
Paracortina carinata : Liu and Tian 2015: 139, key.

##### Diagnosis.

*P.carinata* appears most similar to *P.stimula* especially in the expanded distal part of the telopodite, of which the distal part is downturned and crossing with the curved process of the solenomere, differing in the shape of the distal process, showing as a subrectangular plate in *carinata* vs dome-shaped in *P.stimula*. Both species could be recognised also by the body colouration which is dark brown in *P.carinata* and yellow in *P.stimula*.

##### Descriptive notes.

(based on Wang and Zhang 1993) Holotype 42 mm long, 2.5 mm wide, 60 podous + 2 apodous PTs, with a dark brown colour.

***Gonopods*.** Each gonopod with two asymmetrical prefemoroidal processes (**pf1, pf2**), with **pf1** larger and more setose than **pf2**, a stout and falcate coxal process, reaching 2/3^rd^ of the telopodite; telopodite (**T**) with a uniformly broad stem, distal part abruptly expanding antero-posteriad and showing in a lateral view (Wang and Zhang 1993, fig. 29) a subrectangular plate with an irregular apical margin, and a notch on the anteromesal side separating the main branch from a curved narrow process pointing distad, terminating in a slender branch bifurcated in solenomere (**s**) and parasolenomere (**ps**).

##### Distribution.

Shangrila (= Zhong Dian) County, Yunnan, China (Fig. 28).

##### Comments.

Known only from its original description (Wang and Zhang 1993).

#### ﻿ Paracortina kabaki

Taxon classificationAnimaliaCallipodidaParacortinidae

Akkari & Stoev sp. nov.

3BF11D71-DBA4-58C7-9EE6-198BF5BF3DF1

https://zoobank.org/7F96C628-A9B7-49F3-804E-A993B9D4BAE2

**Figure 8.**
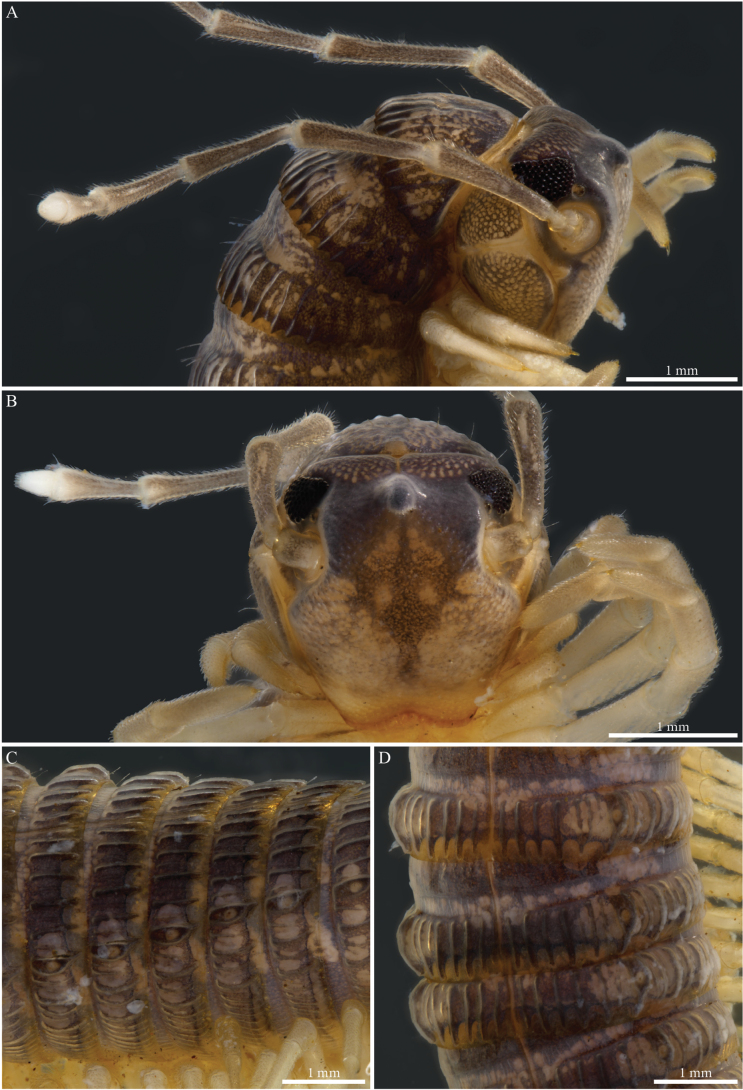
*Paracortinakabaki* Akkari & Stoev, sp. nov., male holotype (Rd 5349 ZMUM) **A, B** head and anterior pleurotergites **A** lateral view **B** frontal view **C, D** midbody pleurotergites **C** lateral view **D** dorsal view.

**Figure 9.**
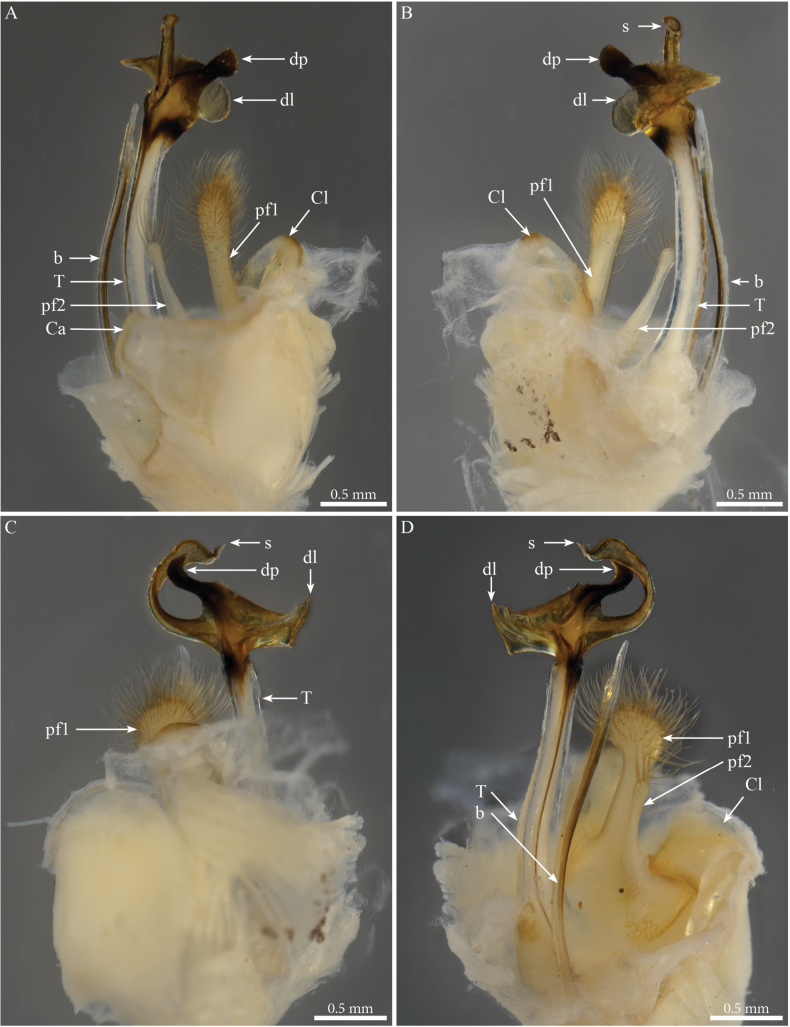
*Paracortinakabaki* Akkari & Stoev, sp. nov., male holotype (Rd 5349 ZMUM), right gonopod **A** anterior view **B** posterior view **C** lateral view **D** mesal view. Abbreviations: b = mesal process of coxa; Ca = anterior lobe of coxa; Cl = lateral lobe of coxa; dl =distal lamella of telopodite; dp = a median projection of the distal part of telopodite; pf1 = prefemoroidal process 1; pf2 = prefemoroidal process 2; s = solenomere; T = telopodite.

**Figure 10.**
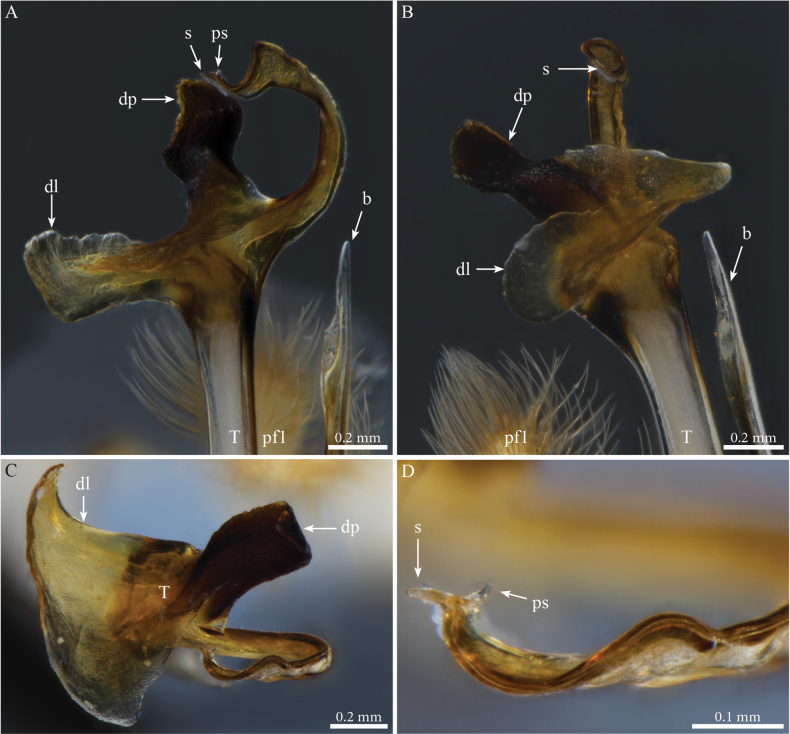
*Paracortinakabaki* Akkari & Stoev, sp. nov., male holotype (Rd 5349 ZMUM) **A–C** right gonopod telopodite, distal part, **A** mesoventral view **B** posteromesal view **C** ventral view **D** detail of solenomere, ventral view. Abbreviations: b = mesal process of coxa 2; dl =distal lamella of telopodite; dp = a median projection of the distal part of telopodite; pf1 = prefemoroidal process 1; ps = parasolenomere; s = solenomere; T = telopodite.

Figs 8 , 9 , 10 , 27E , 28

##### Material examined.

***Holotype***: China, Yunnan, Shangrila County, Degen, 214 Ntn Road, NE slope of SE Baima Mt. Range, between Cukatongcun & Nali, alt. 2465 m, 28°2'23"N, 99°12'16"E, 8.06.2013, I. Belousov, I. Kabak & G. Davidian leg. (Rd 5349 ZMUM); ***Paratype***: 1 male 54PTs, same data as holotype (Rd 5350 ZMUM).

##### Etymology.

The species epithet is a patronym to honours of one of the collectors, Ilya Kabak from the Zoological Institute of the Russian Academy of Sciences St Petersburg. Noun in the genitive case.

##### Diagnosis.

Different from all species of the genus *Paracortina* by the distinctive shape of the distal part of telopodite with the ruffle of distolateral lamella.

##### Description (Holotype).

Body cylindrical, length 77.4 mm, maximal width ca 3.2 mm at PT5; body narrowing anteriorly and posteriorly from PT6; 60 (59 + 1 apodous) pleurotergites (PTs) + telson. Live colour unknown. Preserved specimen with a general dark brown to greyish aspect contrasted with pale legs and antennae (Fig. 8A, B), prozona greyish, sputtered with fine brown dots (Fig. 8A, D); metazona dorsally dark greyish brown especially on crests, anterior part greyish, finely sputtered with pale brown interrupted by larger irregular yellow alveolate spots, colour paler laterally below the ozopores and ventrally; legs yellowish (Fig. 8C, D). Head: frontal part pale brown to yellowish, vertex dark grey-brown, antennae yellow (Fig. 8A, B).

Fields of ommatidia subtriangular, black, composed of ~ 75 transparent ommatidia in nine or ten rows. Organ of Tömösváry large, ~ 1.2 mm, 3 × as large as an ommatidium, situated close to and touching anterior side of eye (Fig. 8A). Antennae long; length of antennomeres (mm): 1 = 0.18; 2 = 1.29; 3 = 1.27; 4 = 1.01; 5 = 1.08; 6 = 0.69; 7 = 0.23.

PTs composed of smooth prozona and carinate metazona, latter being more pronounced in the posterior part. Prozona with no crests, anterior part of metazona with low, fine carinae, posterior part sharply raising forming well-developed longitudinal narrow and subparallel crests, well-separated from one another; crests gradually reduce in size laterally and ventrally (Fig. 8C). Chaetotaxy follows the pattern of all setae being in anterior position on PTs 1–4, setae *b*, *c*, *e* migrating posteriorly on PT5 and all setae posteriorly on PT6 onwards. Crests moderately developed, also on collum, comprising alternating primary and secondary series, primary slightly higher than secondary; collum with ca nine crests on each hemipleurite. Ozopores visible from 6^th^ to 59^th^PT, located on 6^th^ (largest) PT. Hypoproct tripartite, median sclerite largest, subrectangular, bearing a pair of basal macrosetae; lateral sclerites smaller, triangular, with one seta each. Paraprocts divided into large ventral and smaller dorsal sclerites, each pair with a pair of long macrosetae. Spinnerets long and slender, arising from the caudal edge of epiproct and extending well beyond the margins of paraprocts.

##### Male sexual characters.

Head with a pronounced beak-shaped projection covered with minute setae (Fig. 8A, B). Leg-pairs 1 and 2 reduced and more setose than the rest, leg-pair 2 with a small anterior process and posterior opening of the gonopores. Leg-pair 7 with a large cone-shaped and apically rounded mesal process on coxa, a lateral angular margin separated by a notch, an apical tuft of setae on trochanter, prefemur with a strong constriction proximally on the anterior margin, then strongly swollen distally (Fig. 27F). Coxal sacs present (noticeable) on leg-pairs 3–13/16.

***Gonopods*** (Figs 9, 10). Parallel, each gonopod with asymmetrical, clavate prefemoroidal processes: a thin, short (**pf1**) process bearing a few apical setae, and a much larger densely setose (**pf2**) one (Fig. 9A, B); coxa with rounded lobes on the anterior side (**Ca**) and the lateral side (**Cl)** respectively, engulfing the prefemoroidal processes (Fig. 9 A), and a long falcate mesal coxal process (**b**), reaching the distal part of the telopodite (Fig. 9A, B, D). Telopodite (**T**) stout and straight, with a uniformly broad stem expanding distally in a complex apex comprising a large hyaline lamella (**dl**) with a serrated margin extending anterolaterally in a double horizontal ruffle (Fig. 9B), and a darkly pigmented median projection (**dp**) apically folded and pointing distolaterad (Figs 9, 10); a slender mesal process, curved 180 degrees and pointing anteriad, slightly swollen apically (Figs 9C, D, 10A) before further narrowing and bifurcating in solenomere (**s**) and parasolenomere (**ps**).

#### ﻿ Paracortina kyrang

Taxon classificationAnimaliaCallipodidaParacortinidae

Nguyen, Stoev, Nguyen & Vu, 2023

E964AA8D-99C7-5751-A44E-688C7A2F4BCC

Fig. 28

Paracortina kyrang Nguyen, Stoev, Nguyen & Vu, 2023: 183, figs 1–7.

##### Diagnosis.

The gonopods of this species differ from those of the other congeners in the sinuous aspect of the telopodite, distally narrowing, reminding more of the genus *Angulifemur*, from which this species differs by the absence of (**Tp**) process and the presence of a long coxal process (**b**) reaching the distal part of telopodite.

##### Descriptive notes.

(based on Nguyen et al. 2023) Species with 68–74 PTs + telson, general colour pale, living specimens greenish-white, head strongly modified in males with a well-protruding projection, 19–20 ommatidia in two or three rows.

##### Male sexual characters.

PTs 6 and 7 strongly enlarged, leg-pairs 1 and 2 smaller and more setose than the rest, showing femoral and tarsal “brushes”, leg-pair 2 with a small anterior process and posterior gonopore, leg-pair 7 with a round mesal projection and a small spine (Nguyen et al. 2023: fig. 6a, b), coxal sacs present on leg-pairs 3–26.

***Gonopods*.** Each gonopod with two clavate, slender, and setose prefemoroidal processes (**pf1, pf2**), coxa low with anterior and lateral rounded lobes and a long falcate mesal process, reaching the distal part of the telopodital stem. Telopodite (**T**) sinuous bearing a distomesal triangular tooth, further twisted and expanding mesolaterad, then strongly constricted and apically narrowed, terminating in a slender branch bifurcated into solenomere (**s**) and parasolenomere (**ps**).

##### Distribution.

Ky Rang Cave, Quoc Toan commune, Quang Hoa District, Cao Bang Province, Vietnam (Fig. 28).

##### Comments.

Nguyen et al. (2023) described a slightly different chaetotaxy for *Paracortinakyrang* with two pairs of setae distributed posteriorly on the collum (usually all setae are positioned anteriorly) and five pairs of posterior setae on PT5 (instead of three pairs).

#### ﻿ Paracortina leptoclada

Taxon classificationAnimaliaCallipodidaParacortinidae

Wang & Zhang, 1993

2BA9DCE2-4368-50E6-BB70-0DE130566823

Fig. 28

Paracortina leptoclada Wang & Zhang, 1993: 376–377, figs 1–5; Stoev and Geoffroy 2004: 99, 103, key; Liu and Tian 2015: 139, key.

##### Diagnosis.

Most similar to *P.thallina*, with an expanded distal part of the telopodite bearing a large rounded lateral lamella and a hook-shaped process pointing anterodistad. Different in the shape and orientation of the distal lamella, laterally positioned and folded 180 degrees and a shorter mesal coxal process.

##### Descriptive notes.

Male holotype 55 mm long, 2.3 mm wide, general colour brown, 55 podous +2 apodous PTs, coxa of leg pairs 1 and 7 with two processes (Wang and Zhang 1993; Wang 1996), head with a large projection on the vertex (Wang 1996).

##### Male sexual characters.

(based on Wang and Zhang 1993: fig. 19) Leg-pair 7 with two processes on coxa.

***Gonopods*.** Parallel and slightly diverging. Each gonopod with two uniformly setose, clavate prefemoroidal processes (**pf1** and **pf2**), one large falcate coxal process narrowing at mid-length and apically projecting in a pointed tip, reaching the distal part of the telopodite. Telopodite (**T**) with a stout stem, distally expanding in a larger process projecting in a broad lateral folded lamella and a thin curved mesal process pointing anterodistad, apically bifurcated to accommodate the solenomere (**s**) and parasolenomere (**ps**).

##### Distribution.

Shangrila County, Yunnan, China (Fig. 28).

##### Comments.

Species known only from its original description.

#### ﻿ Paracortina multisegmentata

Taxon classificationAnimaliaCallipodidaParacortinidae

Stoev & Geoffroy, 2004

99D7AC7F-C0C6-5C16-A66B-90FE452515C9

**Figure 11.**
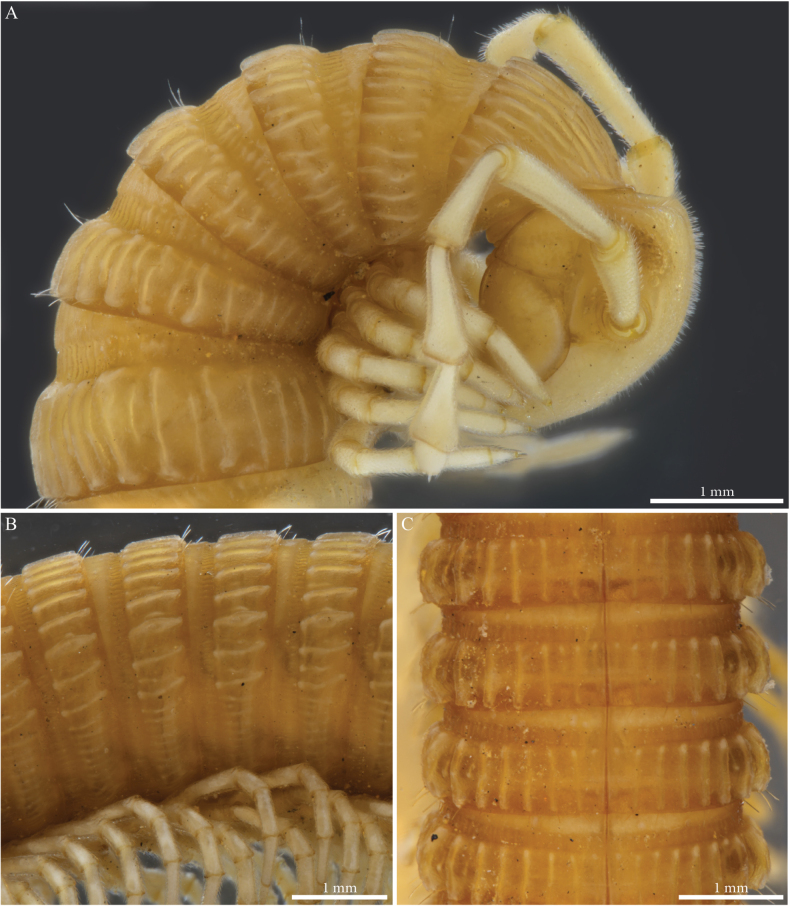
*Paracortinamultisegmentata* Stoev & Geoffroy, 2004, male paratype (BG-NMNHS-INV-000000006261 NMNHS) **A** head and anterior pleurotergites, lateral view **B, C** midbody pleurotergites **B** lateral view **C** dorsal view.

**Figure 12.**
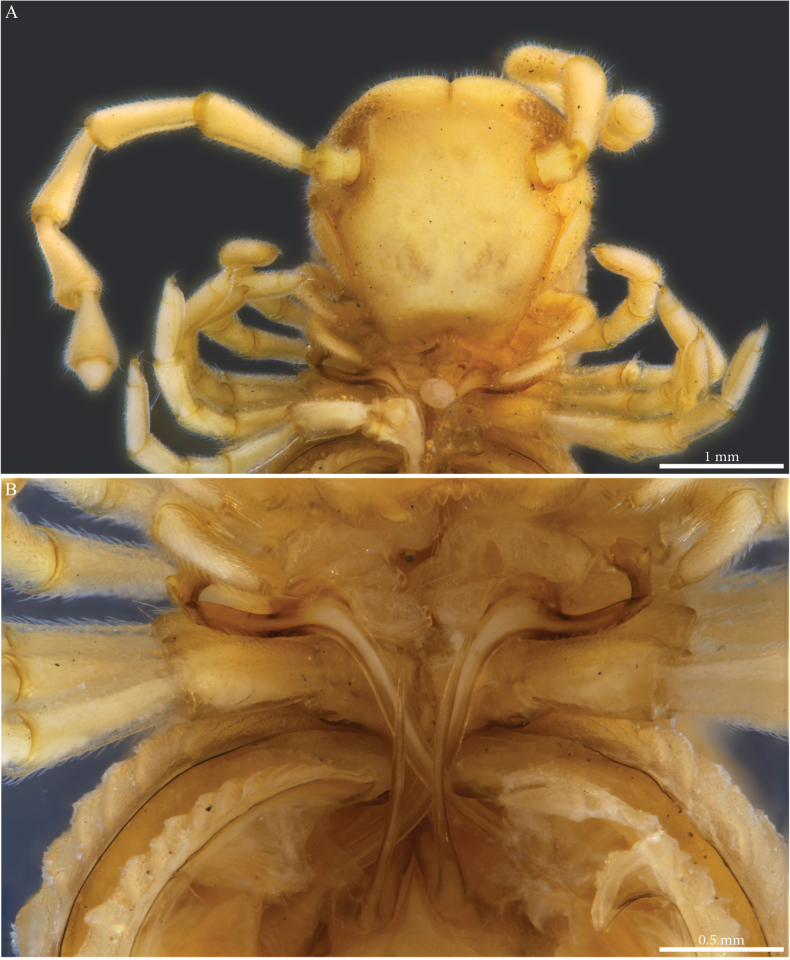
*Paracortinamultisegmentata* Stoev & Geoffroy, 2004, male paratype (BG-NMNHS-INV-000000006261 NMNHS) **A** head, anterior pleurotergites and legs, frontal view **B** gonopods in situ, posterior view.

**Figure 13.**
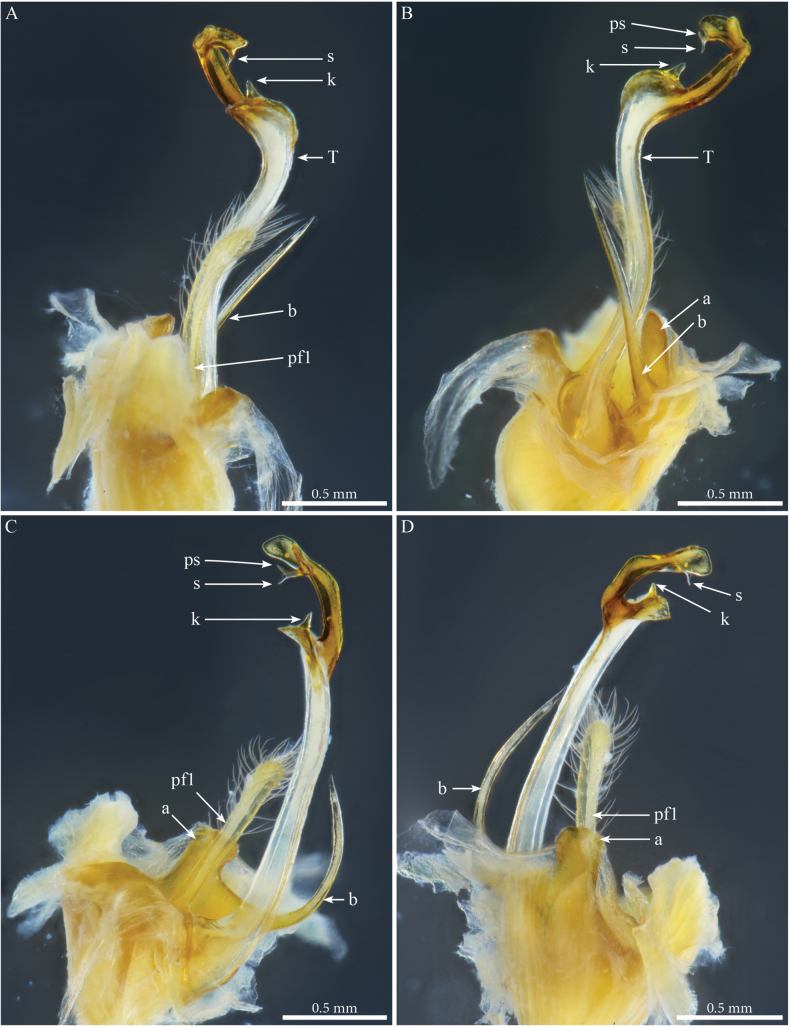
*Paracortinamultisegmentata* Stoev & Geoffroy, 2004, male paratype (BG-NMNHS-INV-000000006261 NMNHS), right gonopod **A** anterior view **B** posterior view **C** lateral view **D** mesal view. Abbreviations: a = mesal coxal lobe; b = coxal falcate process; pf1 = prefemoroidal process 1; k = lateral process of the distal part of telopodite; ps = parasolenomere; s = solenomere; T = telopodite.

Figs 11 , 12 , 13 , 26C , 28

Paracortina multisegmentata Stoev & Geoffroy, 2004: 97, figs 9–17; Liu and Tian 2015: 139, key.

##### Material examined.

1 male paratype, Vietnam, Thanh Hoa Province, Ngọc Lặc, Moc–Trach Cave, alt. 15 m, 8–10.12.1929, Colani leg. (BG-NMNHS-INV-000000006261 NMNHS). Stoev det. April 2004.

##### Diagnosis.

Unique in having the highest number (81–85) of pleurotergites and gonopods with proximally crossing telopodites, distally bent at 90 degrees.

##### Descriptive notes.

Species with 81–85 PTs + telson, general colour of conserved specimens pale brownish (Fig. 11A, B), head convex, unmodified (Figs 11A, 12A), ~ 40 ommatidia in five or six rows (Fig. 11A).

##### Male sexual characters.

PTs 6 and 7 strongly enlarged, leg-pairs 1 and 2 reduced and more setose than the rest, showing prefemoral and tarsal brushes, leg-pair 2 with the posterior gonopore (Fig. 12A, B), leg-pair 7 with a small mesal spine on coxa and a tuft of setae on trochanter (Fig. 26C), coxal sacs present on leg-pairs 3–23.

***Gonopods*** (Figs 12B, 13). Converging, proximally crossing (Fig. 12B). Each gonopod with one slender and distally uniformly setose prefemoroidal process (**pf1**), reaching to overpassing the mid-length of telopodite (Fig. 13); coxa with a mesal rounded lobe (**a**) connected to a slender falcate mesal process (**b**), latter reaching mid-length of the telopodite. Telopodite (**T**) with uniformly slender stem, distally 90 degrees bent laterad (Fig. 12A, B), distal part expanding and terminating in two asymmetrical branches (Fig. 13): a shorter horizontally leaf-like subapical process, surmounted by a triangular tooth (**k**) pointing distolaterad, second branch longer, extending laterad before curving distad, with the apical part terminating in two asymmetrical bulges, the largest accommodating the bifurcated branch with the opening of the solenomere (**s**) and parasolenomere (**ps**).

##### Distribution.

Ngoc-Lac and Loc Thinh, Thanh Hoa, Vietnam (Fig. 28).

##### Comments.

This species possesses unique characters within the family, not only by having the highest number of pleurotergites but also in being the only species of Paracortinidae with telopodites proximally crossing and their distal part being bent to 90 degrees. This combination of characters could justify the description of a new genus to accommodate the species. However, similar to *P.kyrang*, until more material becomes available, we refrain from erecting new genera for these two species and leave them in the genus *Paracortina* until further analyses are available.

#### ﻿ Paracortina serrata

Taxon classificationAnimaliaCallipodidaParacortinidae

(Wang & Zhang, 1993)

A7FAF468-DE7C-500D-ABF3-17633BEAAA4C

Fig. 28

Altum serratum Wang & Zhang, 1993: 383–385, figs 24–28.
Paracortina serrata : Stoev and Geoffroy 2004: 102, key; Liu and Tian 2015: 139, key.

##### Diagnosis.

Most similar to the new species *P.kabaki* sp. nov., especially in the distal part of the telopodite with a large, serrated lamella, differing by the short trapezoid lamella (vs larger rectangular one).

##### Descriptive notes.

Holotype 45 mm long, 2.9 mm wide, 52 podous + apodous PTs, general colour brown.

##### Male sexual characters.

Leg-pair 7 with two coxal processes (Wang and Zhang 1993; Wang 1996). ***Gonopods*** (based on Wang and Zhang 1993: figs 24–26). Parallel, each gonopod with two asymmetrical, clavate prefemoroidal processes, with (**pf1**) larger and more setose than (**pf2**), the latter distally folded; one large broad and long coxal process (**b**), reaching the distal part of the telopodite and apically projecting in a pointed tip. Telopodite (**T**) with a stout stem, distally expanding into a lateral lamella with a serrated margin, separated by a deep notch from a median branch. Latter presenting a blunt subapical projection, and a longer twisted process, curved and bifurcating at the tip, with the opening of the solenomere (**s**) and parasolenomere (**ps**).

##### Distribution.

Deqin County, Yunnan, China (Fig. 28).

##### Comments.

Species known only from its original description.

#### ﻿ Paracortina stimula

Taxon classificationAnimaliaCallipodidaParacortinidae

(Wang & Zhang, 1993)

480B083F-CC9D-537A-9F6C-F67D72AFF1BE

Fig. 28

Relictus stimulus Wang & Zhang, 1993: 379–380, figs 10–13.
Paracortina stimula : Stoev and Geoffroy 2004: 102, key; Liu and Tian 2015: 139, key.

##### Diagnosis.

Most similar to *P.carinata*, differing in the distal process, being dome-shaped (vs sub rectangular in *P.carinata*). Both species could be recognised also by the body colouration which is dark brown in *P.carinata* and yellow in *P.stimula*.

##### Descriptive notes.

Holotype 35.5 mm long, 1.9 mm wide, number of PTs in holotype not specified, in female allotype 53 podous + 3 apodous; general colour light yellow (Wang and Zhang 1993).

***Gonopods*** (based on Wang and Zhang 1993: figs 10, 11). Each gonopod with two asymmetrical clavate prefemoroidal processes with (**pf1**) large and setose, (**pf2**) small with only a pair of large setae; one large falcate coxal processes (**b**) reaching the distal part of the telopodite, apically projecting in a pointed tip. Telopodite (**T**) with a stout stem, distally expanding into dome-like plate, with a notch on the mesal margin separating two oppositely directed crossing projections of more or less similar length: a downturned projection and upturned process pointing distomesad, bifurcated at tip with the opening of the solenomere (**s**) and parasolenomere (**ps**).

##### Distribution.

Shangrila (= Zhong Dian) County, Yunnan, China (Fig. 28).

##### Comments.

Species known only from its original description.

#### ﻿ Paracortina thallina

Taxon classificationAnimaliaCallipodidaParacortinidae

(Wang & Zhang, 1993)

2C7AE4DB-7B8F-5334-9693-F44B84586552

**Figure 14.**
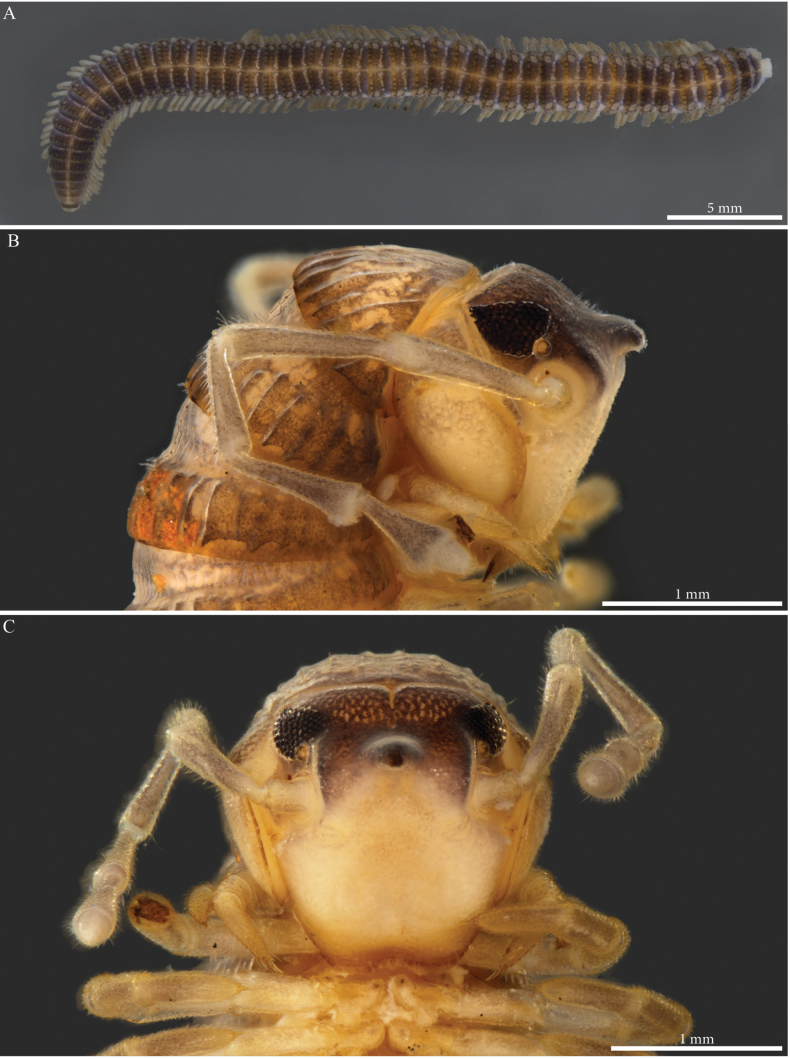
*Paracortinathallina* (Wang & Zhang, 1993), male (Rd 5344 ZMUM) **A** habitus (head missing), dorsal view **B, C** head and anterior pleurotergites **B** lateral view **C** frontal view.

**Figure 15.**
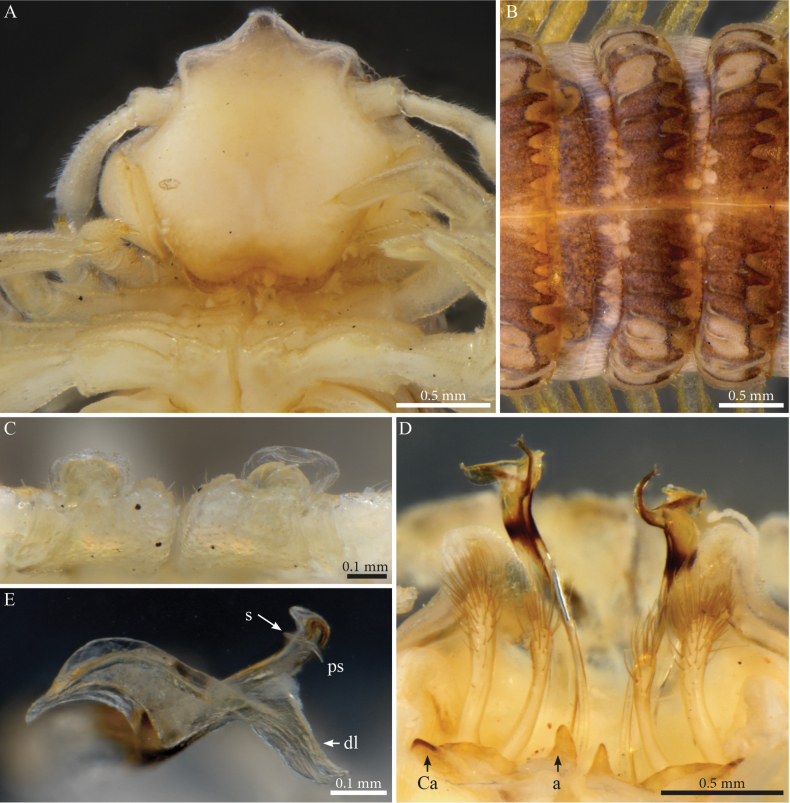
*Paracortinathallina* (Wang & Zhang, 1993) male (Rd 5344 ZMUM) **A** head and anterior pleurotergites and legs, frontal view **B** midbody pleurotergites, dorsal view **C** 9^th^ leg pair coxal sacs, posterior view **D** gonopods, anterior view **E** right telopodite, apical section, ventral view. Abbreviations: a = mesal anterior triangular process of coxa; Ca = anterior lobe of coxa; dl =distal lamella of telopodite; ps = parasolenomere; s = solenomere.

**Figure 16.**
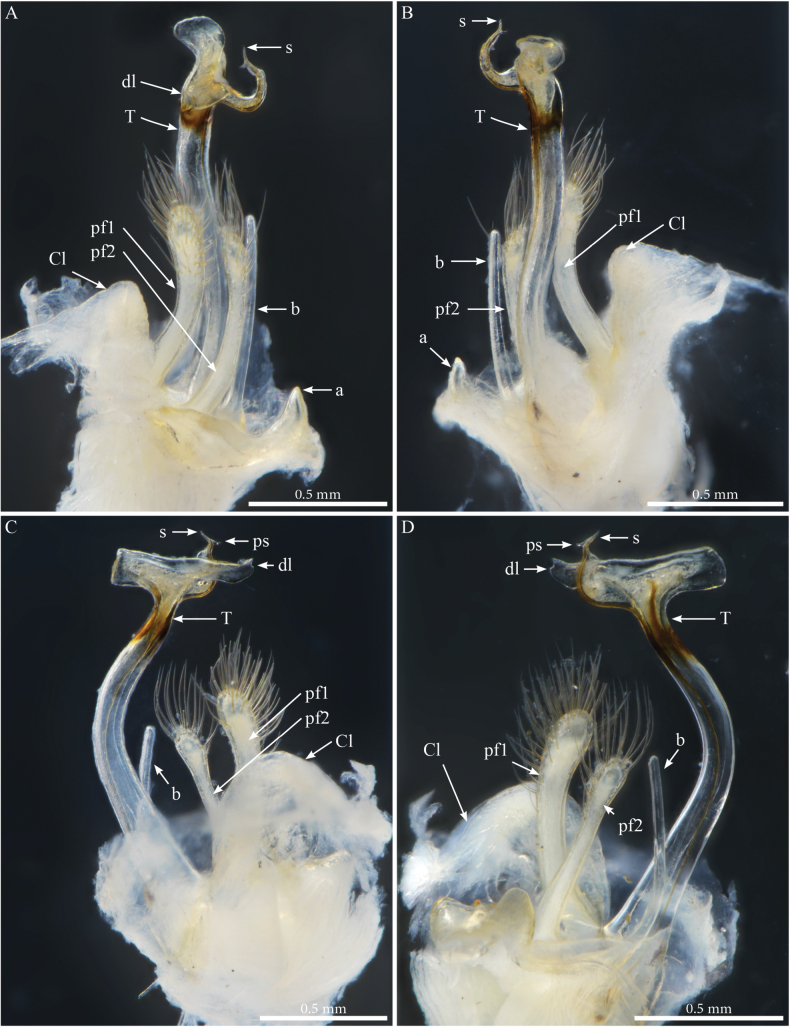
*Paracortinathallina* (Wang & Zhang, 1993), male (Rd 5344 ZMUM), right gonopod **A** anterior view **B** posterior view **C** lateral view **D** mesal view. Abbreviations: a = mesal anterior triangular process of coxa; b = falcate mesal process of coxa; Cl = lateral lobe of coxa; dl =distal lamella of telopodite; pf1 = prefemoroidal process 1; pf2 = prefemoroidal process 2; ps = parasolenomere; s = solenomere; T = telopodite.

Figs 14 , 15 , 16 , 26D, E , 28

Relictus thallinus Wang & Zhang, 1993: 380, figs 14–18.
Paracortina thallina : Stoev and Geoffroy 2004: 103, key; Liu and Tian 2015: 139, key.

##### Material examined.

1 male, China, Yunnan, Shangrila, Degen, SW of Benzibanzhen, 214 Nm Road, NE slope of SE Baima Mt. Range, 28°6'50"N, 99°12'24"E, alt. 3260, 07.06.2013, I. Belousov, I. Kabak & G. Davidian leg. (Rd 5344 ZMUM), Stoev & Akkari det. 2023.

##### Diagnosis.

Most similar to *P.leptoclada* in the expanded distal part of the telopodite bearing a large rounded lateral lamella and a hook-shaped process pointing anterodistad. Different in the shape and orientation of the distal lamella, (vs laterally expanded and folded lamella and a shorter mesal coxal process in *P.leptoclada*).

##### Descriptive notes.

Male with 55 PTs + Telson. Length ca 39.7 mm. Live colour unknown. Preserved specimen with a general dark brown to greyish colour, legs and antennae yellowish with a dark sputter (Figs 14, 15A), prozona greyish with fine brown sputter (Figs 14A, 15B); metazona dark greyish brown dorsally especially on crests, anterior part greyish with a fine pale brown sputter interrupted by larger irregular yellow alveolate spots, colour gradually fading below the ozopores and ventrally. Head with dark pigmentation on the vertex and frons, mandibular stipes and gnathochilarium yellowish (Fig. 14B, C). Fields of ommatidia subtriangular, composed of ~ 62 ommatidia in ten rows. Organ of Tömösváry very large, ~ 0.8 mm, situated close to and touching the anterior side of eye (Fig. 14B).

##### Male sexual characters.

Head with a triangular protruding projection on vertex (Figs 14B, C, 15A). Leg-pairs 1 and 2 reduced and setose (Fig. 14C), showing prefemoral and tarsal brushes, leg-pair 2 with a dome-shaped anterior process on coxa and posterior gonopore, leg-pair 4 with an anterior triangular process on coxa (Fig. 15A), leg-pair 6 with a small mesal tooth on coxa (Fig. 26D), leg-pair 7 with one mesal and one lateral slender hyaline pointed processes on coxa (Fig. 26E), coxal sacs (Fig. 15C) noticeable from leg-pair 3–23.

***Gonopods*** (Figs 15D, E, 16). Diverging. Each gonopod with two large, setose, clavate prefemoroidal processes (**pf1**, **pf2**) with **pf1** slightly larger than **pf2** (Fig. 15D); coxa with a large lobe laterally (**Cl**) and a low projection (**Ca**) anteriorly (Figs 15D, 16), a mesal anterior triangular process (**a**) and a long falcate coxal processes (**b**) reaching the distal part of the telopodite (both broken in the studied specimen). Telopodite (**T**) with a long slightly curved stem, distally abruptly expanding in a sub-rectangular posterior process with rounded margins (Fig. 16C, D) connected to a transparent jagged lamella (**dl**) projecting anteriad (Fig. 15D), apically folded (Fig. 16C, D), and a curved hook-shaped process pointing meso-anteriad (Fig. 16), narrowing towards its apex and bifurcating into the opening of the solenomere (**s**) and parasolenomere (**ps**).

##### Distribution.

Batang County, Sichuan, and Shangrila County, Yunnan, China (Fig. 28).

##### Comments.

This is the second record of the species since its original description. Here we provide the first micrographs illustrating the habitus of the species (Figs 14, 15).

#### ﻿ Paracortina viriosa

Taxon classificationAnimaliaCallipodidaParacortinidae

(Wang & Zhang, 1993)

781E10B1-A876-530E-B0BC-98E21608984E

**Figure 17.**
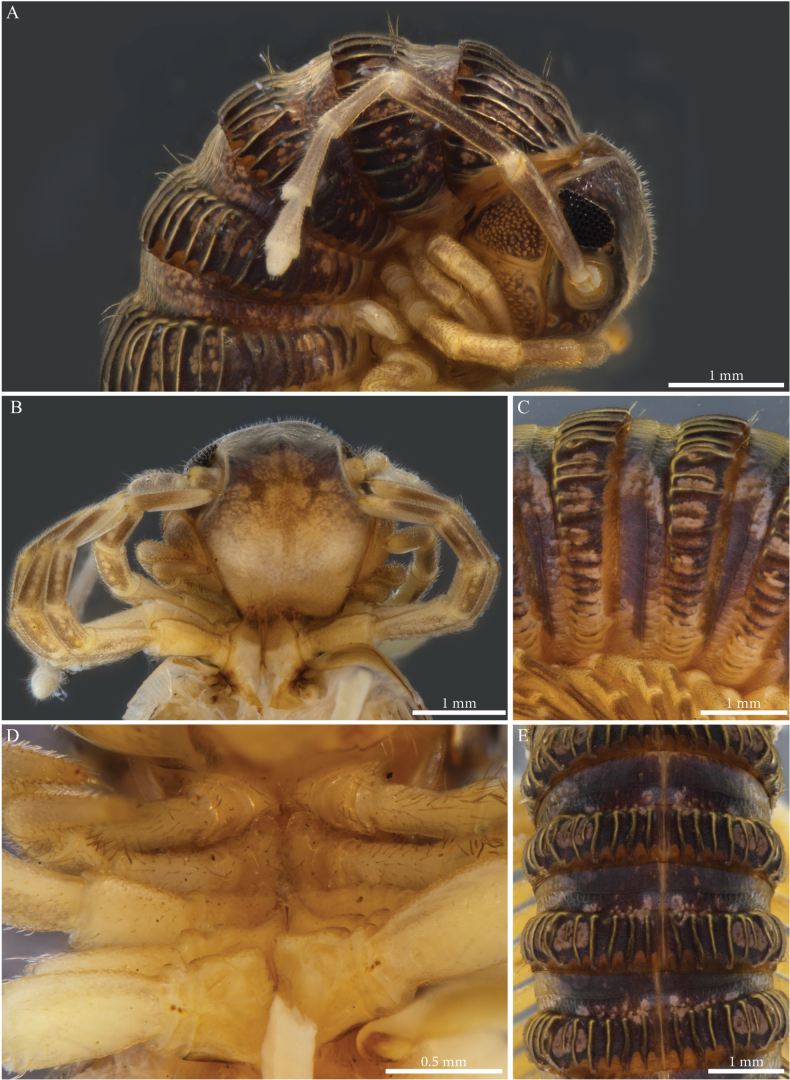
*Paracortinaviriosa* (Wang & Zhang, 1993), male (Rd 5345 ZMUM) **A, B** head and anterior pleurotergites **A** lateral view **B** frontal view **C, E** midbody pleurotergites **C** lateral view **D** leg-pairs 1-5 posterior view **E** dorsal view.

**Figure 18.**
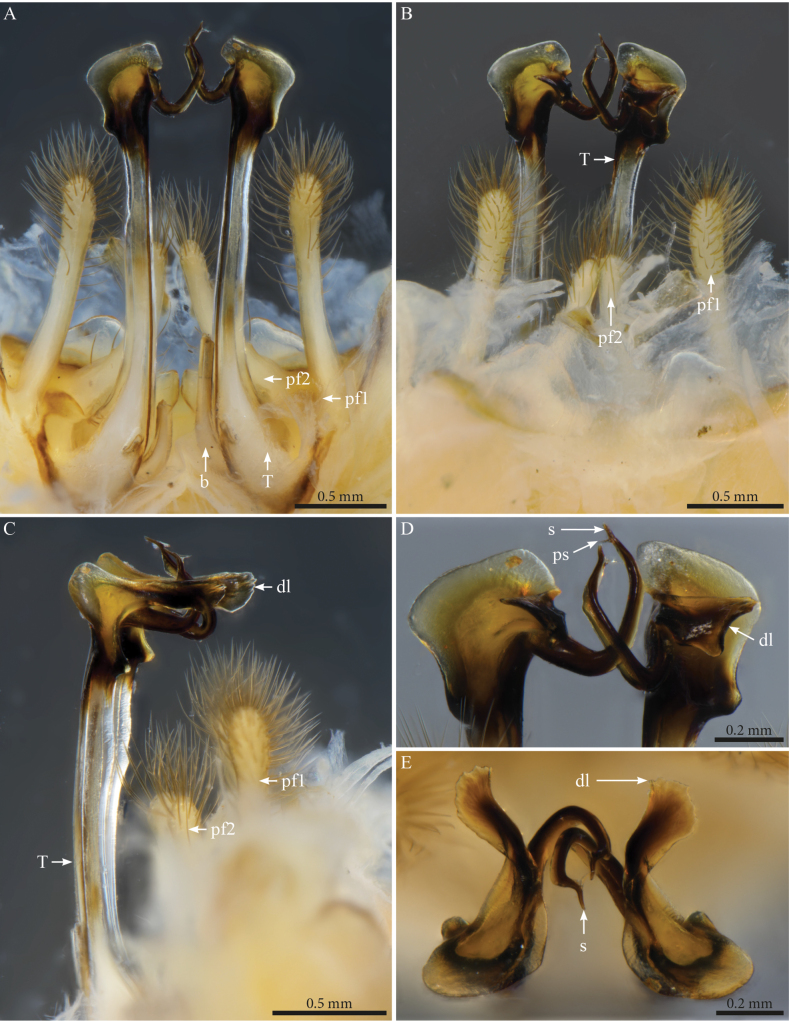
*Paracortinaviriosa* (Wang & Zhang, 1993), male (Rd 5345 ZMUM), gonopods **A** posterior view **B** anterior view **C** lateral view **D, E** telopodites distal part, **D** anterior view **E** ventral view. Abbreviations: b = falcate mesal process of coxa; dl =distal lamella of telopodite; pf1 = prefemoroidal process 1; pf2 = prefemoroidal process 2; ps = parasolenomere; s = solenomere; T = telopodite.

**Figure 19.**
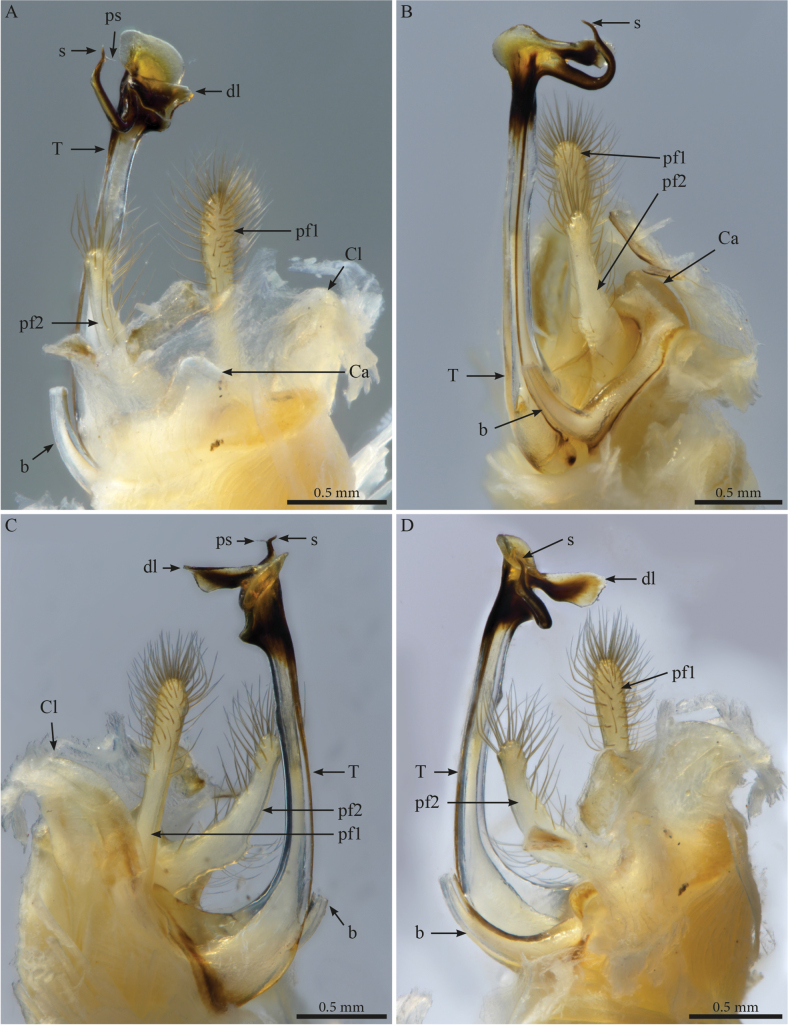
*Paracortinaviriosa* (Wang & Zhang, 1993), male (Rd 5345 ZMUM), right gonopod **A** anterior view **B** posteromesal view **C** lateral view **D** mesal view. Abbreviations: b = falcate mesal process of coxa (broken); Ca = anterior lobe of coxa; Cl = lateral lobe of coxa; dl =distal lamella of telopodite; pf1 = prefemoroidal process 1; pf2 = prefemoroidal process 2; ps = parasolenomere; s = solenomere; T = telopodite.

Figs 17 , 18 , 19 , 27A, B , 28

Altum viriosum Wang & Zhang, 1993: 381, figs 19–23.
Paracortina viriosa : Stoev and Geoffroy 2004: 103, key; Liu and Tian 2015: 139, key.

##### Studied material.

1 male, China, W Lijiang, W Yangtze, W Xinhuacun, 0.7 km NW Daqingtou, 26°56'23"N, 99°52'16"E, 2720m, 01.06.2018, I. Belousov & I. Kabak leg. (Rd 5354 ZMUM), Akkari det. 2023, 2 males, China, NW Lijiang, W Chang J. NW Jinzhuang, 2.5 km N Tuozhi Vill, 27°09'32"N, 99°41'47"E, 2315 m, 17.05.2017, I. Belousov & I. Kabak leg. (Rd 5345 ZMUM), Akkari det. 2023.

##### Diagnosis.

Most similar to *P.voluta* especially in the distal part of the telopodite with two main folds and a strong hook-shaped median processes, but differs from the latter by the shape of the distal process, in posterior view subquadrate with a shallow notch (vs earlobe-shaped), the shape and position of the anterior lamella (at the basis of the distal part, rounded and serrated on the lower margin vs more distal and smaller).

##### Descriptive notes.

Male with 68 PTs + telson. Length ca 60.5 mm. Live colour unknown. Preserved specimen with a general tawny dark brown, legs and antennae with a slightly fading colour but showing hints of dark sputter (Fig. 17A), prozona dark brown with paler alveolate spots (Fig. 17C, E); metazona dorsally dark brown, especially on crests, interrupted by larger irregular yellow alveolate spots on the lateral crests; colour gradually fading below the ozopores and ventrally. Head with dark pigmentation on the vertex and frons, mandibular stipes and gnathochilarium with yellowish spots, labral area paler (Fig. 17A, B). Fields of ommatidia subtriangular, composed of ~ 60 ommatidia in eight rows. Organ of Tömösváry large, situated close to and touching, anterior side of eye (Fig. 17A).

##### Male sexual characters.

Head with a small projection on vertex (Fig. 17B). Leg-pairs 1 and 2 reduced and more setose than the rest, showing prefemoral and tarsal brushes (Fig. 17B), leg-pair 2 with a large dome-shaped anterior process on coxa and posterior gonopore (Fig. 17D), leg-pairs 3 and 4 with anterior triangular projections on coxa (Fig. 17D), leg-pair 6 with a small rounded mesal projection on coxa and a slight constriction of prefemur proximally on the posterior margin (Fig. 27A), leg-pair 7 with strongly modified coxa, anteriorly projecting in a large mesal slightly curved horn (Fig. 27B), trochanter with strong setae.

***Gonopods*.** Parallel, distal solenomeral processes of telopodites crossing (Fig. 18A, B, D, E). Each gonopod with two asymmetrical clavate prefemoroidal processes, with **pf1** broader and more setose than **pf2** (Figs 18A, B, 19); coxa with a large rounded anterior lobe (**Ca**) and a smaller lateral one (**Cl**) (Fig. 19A); long and falcate mesal coxal process (**b**), reaching the distal part of the telopodite and distally projecting in a pointed tip (broken in the studied specimen). Telopodite (**T**) with a long and uniformly broad stem, distally abruptly expanding in an apically folded subquadrate plate, with, in posterior view, a slightly oblique and indented lateral margins (Fig. 18A), in anterior view (Fig. 18B) showing a darkly pigmented jagged lamella (**dl**) projecting antero-laterad (Fig. 18C–E), and a strong hook-shaped curved process pointing distad, gently narrowing towards its apex (Figs 18, 19) and bifurcating into the opening of the solenomere (**s**) and parasolenomere (**ps**).

##### Distribution.

Shangrila and Lijiang County, Yunnan, and Mangkang/Markam? County, Tibet Autonomous Region, China (Fig. 28).

##### Comments.

The structures on the tip of the falcate mesal coxal process of the gonopod were also illustrated in the original description of *P.viriosa* by Wang and Zhang (1993) although never mentioned in the description of the species (Wang and Zhang 1993: 382, fig. 21).

#### ﻿ Paracortina voluta

Taxon classificationAnimaliaCallipodidaParacortinidae

Wang & Zhang, 1993

1033BE23-414C-5177-9C61-A48CC0360086

**Figure 20.**
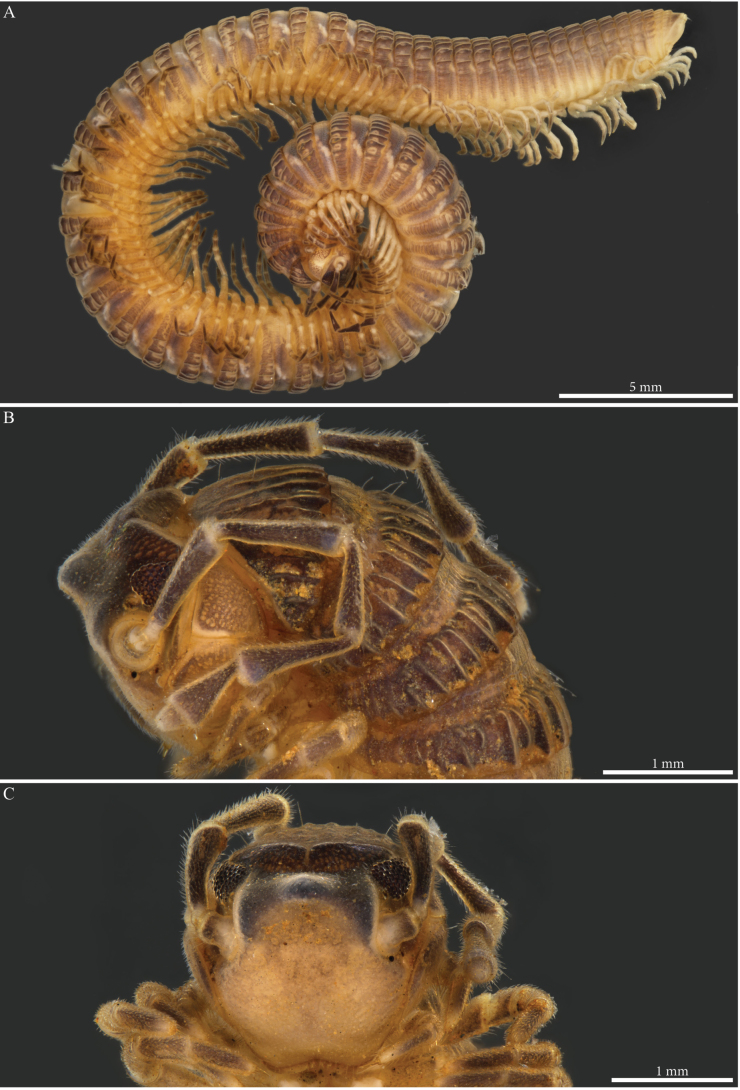
*Paracortinavoluta* Wang & Zhang, 1993 (Rd 5346 ZMUM) **A** female habitus, lateral view **B, C** male head and anterior pleurotergites **B** lateral view **C** frontal view.

**Figure 21.**
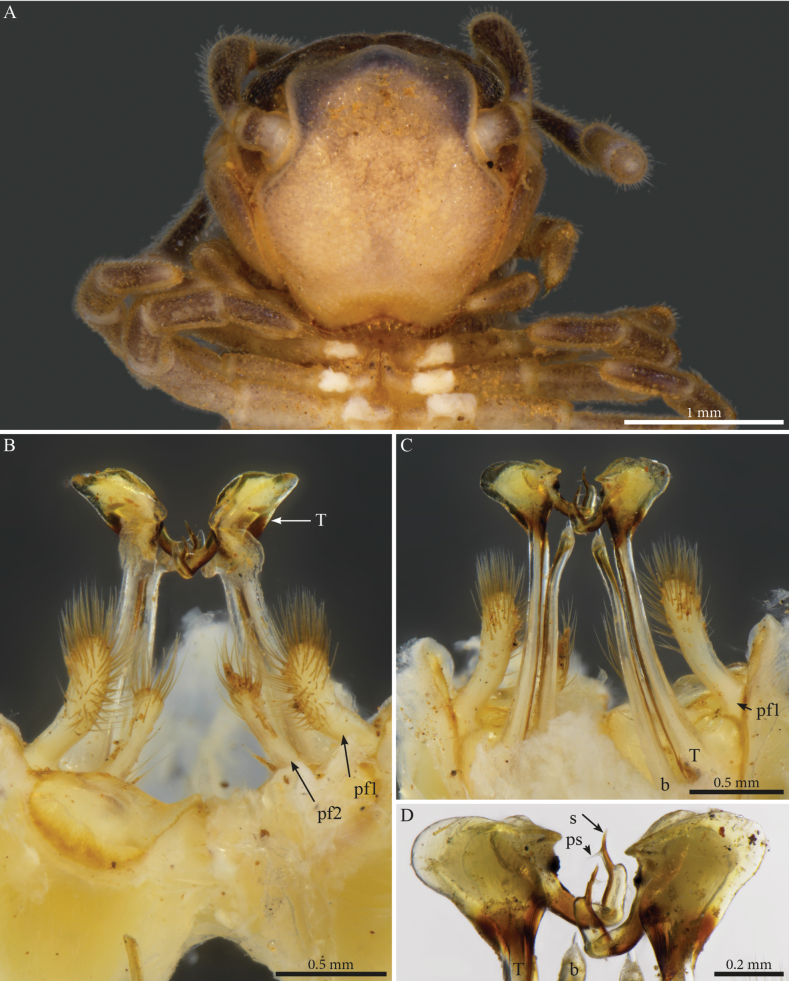
*Paracortinavoluta* Wang & Zhang, 1993, male (Rd 5346 ZMUM) **A** head, anterior pleurotergites and legs, frontal view **B–D** gonopods **B** anterior view **C** posterior view **D** telopodite distal part. Abbreviations: b = falcate mesal process of coxa 2; pf1 = prefemoroidal process 1; pf2 = prefemoroidal process 2; ps =parasolenomere; s = solenomere; T = telopodite.

**Figure 22.**
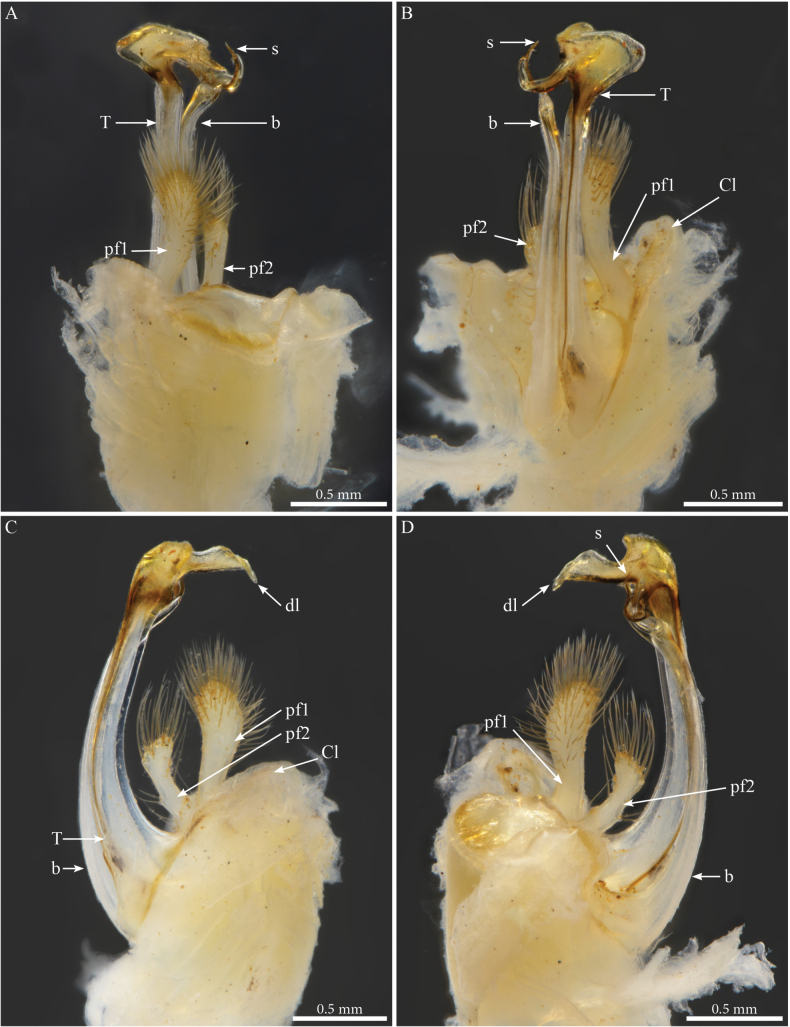
*Paracortinavoluta* Wang & Zhang, 1993, male (Rd 5346 ZMUM), left gonopod **A** anterior view **B** posterior view **C** lateral view **D** mesal view. Abbreviations: b = falcate mesal process of coxa; Cl = lateral lobe of coxa; dl =distal lamella of telopodite; pf1 = prefemoroidal process 1; pf2 = prefemoroidal process 2; s = solenomere; T = telopodite.

Figs 20 , 21 , 22 , 27C, D , 28

Paracortina voluta Wang & Zhang, 1993: 377, figs 6–9; Stoev and Geoffroy 2004: 103, key; Liu and Tian 2015: 139, key.

##### Studied material.

2 males, 1 female, China, Sichuan Province, NW Pingchuan, 27°40'07"N, 101°44'04"E, 18.07.2011, I. Belousov & I. Kabak leg. (Rd 5346 ZMUM), Akkari det. 2023.

##### Diagnosis.

Most similar to *P.viriosa* in the shape of the distal part of the telopodite with two main folds and a strong hook-shaped median processes, differing in the earlobe-shaped distal process and the smaller and more distally located anterior lamella.

##### Descriptive notes.

Male with 56PTs+Telson. Length ca 49.5 mm. Live colour unknown. Preserved specimen with a brownish general colour, legs and antennae dark brown (Figs 20, 21A), Head with dark pigmentation on the vertex and frons (Fig. 20B, C), mandibular stipes and gnathochilarium with yellowish spots. Fields of ommatidia subtriangular, composed of ~ 47 ommatidia in ten rows (Fig. 20B, C). Organ of Tömösváry very large, ~ 1.2 mm, situated close to and touching anterior side of eye (Fig. 20B). Prozona brownish sputtered with a paler colour; metazona dorsally dark tawny-brown, especially on crests, anterior part pale with brown alveolate spots (Fig. 20A, B).

##### Male sexual characters.

Head with a protruding triangular projection on vertex (Fig. 20B, C, 21A). Leg-pairs 1 and 2 reduced and more setose than the rest, showing prefemoral and tarsal brushes, leg-pair 2 with a large anterior process on coxa and posterior gonopore (Fig. 21A), leg-pair 4 with anterior triangular projection on coxa (Fig. 21A), leg-pair 6 with one short triangular mesal process and a smaller lateral one on coxa, prefemur proximally slightly constricted on the posterior margin (Fig. 27C), leg-pair 7 with one mesal hyaline pointed process and a rounded projection surmounted by a similar but slightly smaller one, trochanter with a tuft of strong setae (Fig. 27D).

***Gonopods*.** Parallel, slightly converging with the distal solenomeral processes of telopodites crossing (Fig. 21B–D). Each gonopod with two asymmetrical, short, clavate prefemoroidal processes with (**pf1**) broader and more setose than (**pf2**) (Figs 21B, C, 22); coxal anterior lobe lower than the lateral lobes low (Fig. 21C), long and falcate coxal process (**b**) reaching the distal part of the telopodite and apically pointed (Figs 21C, 22A, B, D). Telopodite (**T**) with a long stem, distally expanding in an earlobe shape with rounded lateral margin seen in posterior view (Fig. 21C, D), in anterior view as an oblique subtrapezoidal plate (Fig. 21B), with a transparent lamella (**dl**) attached on the lower part, marking and S-shape and serrated on the lower margin (Fig. 21B), mesal process curved upward, twisted and narrowing towards its apex (Figs 21B–D, 22A, B), bifurcating into the opening of the solenomere (**s**) and parasolenomere (**ps**).

##### Distribution.

This species was originally described from Ya Jang (= Jajiang) County, Sichuan, China. Here we add a new record from Yanyuan County which is ~ 250 km away in a straight line from the type locality (Fig. 28).

##### Comments.

Although we have no doubt about the identity of the studied specimens, when comparing the gonopod of the new material in mesal view (Fig. 20D) with the drawing provided in the original description of *P.voluta* (Wang & Zhang, 1993: fig. 6), we noticed a few differences in the shape of the distal process of the telopodite, with the mesal process more twisted and curved, the anterior lamella more serrated and turned downwards, and the “s-twist” more obvious in our specimen (visualised with a rounded notch in mesal view).

Both available identification keys for the family Paracortinidae (Stoev and Geoffroy 2004; Liu and Tian 2015) mention eight or nine “macrosetae” on the small prefemoroidal process and use this character to separate the species from its congener *P.leptoclada*. However, in the specimens we examined, this number greatly exceeds that (see Figs 21, 22), proving that this character is not reliable for species discrimination.

#### ﻿ Paracortina yinae

Taxon classificationAnimaliaCallipodidaParacortinidae

Liu & Tian, 2015

A6646EDB-350B-5F62-A124-12F83A4C48A7

Fig. 28

Paracortina yinae Liu & Tian, 2015: 131, figs 23–45.

##### Diagnosis.

Gonopods very similar to those in *Paracortinazhangi*. The species, can, however, be recognised by the presence of a spiniform mesal process on coxa 6, and a different shape of the coxa of leg 7 (Liu and Tian 2015).

##### Descriptive notes.

(after Liu and Tian 2015) Species with 53–61 PTs + telson, general colour pale brownish yellow, head with a well protruding beak-shaped projection, ommatidia 21–32 in four irregular rows.

##### Male sexual characters.

Head with a small beak-shaped projection on vertex (Liu and Tian 2015: fig. 23), PT6 strongly enlarged, leg-pairs 1 and 2 reduced and more setose than the rest, leg-pair 2 with a small anterior process and posterior opening of the gonopores, leg-pair 6 with a small pointed mesal process on coxa, leg-pair 7 with a mesal slender and pointed triangular process and a large rounded projection on coxa (Liu and Tian 2015: figs 30, 31), coxal sacs noticeable on leg-pairs 3–25.

***Gonopods*.** Parallel, with a general slender aspect. Each gonopod with one prefemoroidal process that is clavate, slender, and setose (**pf1**), coxa with an anterior lobe and a short falcate mesal process (**b**) not reaching mid-length of the telopodite. Telopodite (**T**) broad at the base, gently narrowing distad, distal part dark with a lamella extending in a subtriangular fold, apically blunt and almost rounded, subapically more acuminate and the base slightly uneven, and a short slender bifurcated process, terminating in solenomere (**s**) and parasolenomere (**ps**).

##### Distribution.

Cave I, 24.875732°N, 105.150143°E, Yanchang Village, Tianshengqiao Town, Longlin County, Guangxi, South China (Fig. 28).

##### Comments.

Very similar to *P.zhangi* (see below), differing mostly in the presence of small, pointed, mesal processes on coxae 6 in males and different shape of the coxa on leg-pair 7 (Liu and Tian 2015). The gonopods of the two species are strikingly similar but an accurate comparison is virtually impossible as the authors did not illustrate the gonopods in the exact same view.

#### ﻿ Paracortina zhangi

Taxon classificationAnimaliaCallipodidaParacortinidae

Liu & Tian, 2015

C5519453-315B-52A7-B7EC-24C174177507

Fig. 28

Paracortina zhangi Liu & Tian, 2015: 125, figs 1–22.

##### Diagnosis.

Most similar to *Paracortinayinae*, from which it differs only in the absence of the spiniform process on the mesal side of coxa 6, different shape of coxa 7 (Liu and Tian 2015).

##### Descriptive notes.

(based on Liu and Tian 2015) Species with 55–58 PTs +telson, general colour pale brownish yellow, head with a well protruding beak-shaped projection, ommatidia: 16–23 in four irregular rows.

##### Male sexual characters.

Head with a large beak-shaped projection on vertex (Liu and Tian 2015: figs 1, 2), PT6 strongly enlarged, leg-pair1 and 2 reduced and more setose than the rest, leg-pair 2 with a small anterior process and posterior opening of the gonopores, leg-pair 6 with no modifications on coxa, leg-pair 7 with a protruding mesal triangular process and a very large subtriangular-rounded projection on coxa (Liu and Tian 2015: fig. 8), coxal sacs noticeable from leg-pairs 3–23.

***Gonopods*.** Parallel, with a general slender aspect. Each gonopod with one clavate, slender, setose prefemoroidal process (**pf1**), coxa with an anterior lobe and a short falcate mesal process (**b**), not reaching mid-length of the telopodite (**T**). Telopodite broad at the base and gently narrowing distad, distal part dark with a transparent lamella (seen in lateral view; Liu and Tian 2015: fig. 9) extending in an apical fold, and laterally in a second one circling in part of the thin bifurcated branch terminating in solenomere (**s**) and parasolenomere (**ps**).

##### Distribution.

Cave Qiaoxia Dong 24°03.008'N, Rongdu Village, Qianxinan Zizhizhou, Ceheng County, Guizhou, southern China. The coordinates in Liu and Tian (2015) include a typo (24°03.008'N instead of 25°03.008'N), rendering the type locality appearing 100 km south of their locality description and map. In the distribution map (Fig. 28), we used the corrected coordinates.

##### Comments.

The interpretation of the distal part of the telopodite remains tentative and rendered difficult as it is entirely based on the original description of the leg-pair 7 and gonopods (Liu and Tian 2015: figs 8–10, 15–18).

#### ﻿Genus Scotopetalum

Taxon classificationAnimaliaCallipodidaParacortinidae

Shear, 2000 stat. rev.

92051139-765C-5B19-BF64-F486285B59D9

Scotopetalum Shear, 2000: 96, fig. 1; Stoev and Geoffroy 2004: 94, figs 1–8 (proposed synonymy with genus Paracortina).

##### Type species.

*Scotopetalumwarreni* Shear, 2000.

##### Included species.

*Scotopetalumchinensis* (Stoev & Geoffroy, 2004), comb. nov.; *Scotopetalumwarreni* Shear, 2000.

##### Diagnosis.

Differs from *Angulifemur* by having parallel stems of telopodites; from *Paracortina* by the absence of large anteromedian subfalcate coxal process, and from the genus *Crassipetalum* gen. nov. by the much smaller prefemoroidal process/es and the very reduced subfalcate coxal process (**b**).

##### Comments.

Shear (2000) described the genus *Scotopetalum* in the family Schizopetalidae, with the following diagnosis: “distinct from other genera of Schizopetalidae in lacking any indication of a sternum or coxal process in the gonopod, and in having no crest transition (full number of primary crests present on all segments). Each hemipleurite bears a series of five setae; all are in anterior position on segments 1–4, setae *b*, *d*, and *e* migrating posteriorly on segment 5, and all setae are posterior on segment 6.” The author doubted the validity of family Paracortinidae, which he believed could only have a status of subfamily within Schizopetalidae. He also interpreted the long falcate process typical for the family to be of a sternal origin.

In their review of the family, Stoev and Geoffroy (2004) described two new species of Paracortinidae from China and Vietnam, with the Chinese species (*Paracortinachinensis*) being quite similar to the species previously described by Shear (2000). The authors correctly noted this fact and compared the two species, highlighting the characters that distinguish them. They also synonymised *Scotopetalum* with *Paracortina*, considering the absence of a falcate coxal process and a second prefemoroidal process to be variable characters in the family. However, Stoev and Geoffroy (2004) failed to compare the structure of the telopodite in detail, which is quite specific in these two species. After a careful analysis, and now having much more material for comparison (including some of the *Paracortina* species described by Wang and Zhang 1993), we believe that *Scotopetalum* is a clearly defined morphological group and here we revive its original status.

#### ﻿ Scotopetalum chinensis

Taxon classificationAnimaliaCallipodidaParacortinidae

(Stoev & Geoffroy, 2004) comb. nov.

D1158990-260A-5B3F-8CE1-84C99F3A540F

**Figure 23.**
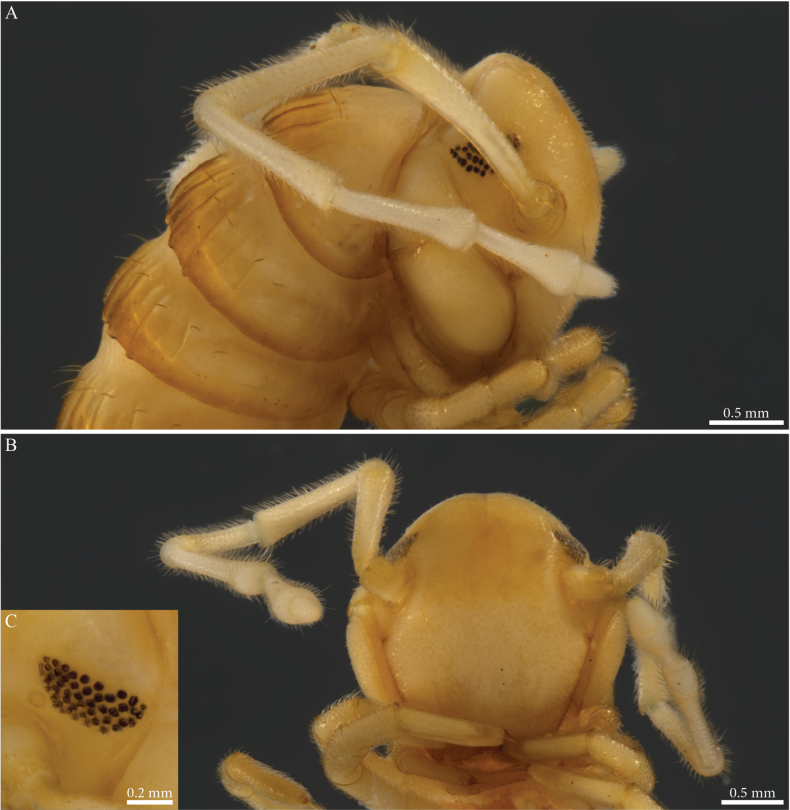
*Scotopetalumchinensis* (Stoev & Geoffroy, 2004), comb. nov., male paratype (BG-NMNHS-INV-000000006262 NMNHS) **A, B** head and anterior pleurotergites **A** lateral view **B** frontal view **C** close-up of ommatidia and organ of Tömösváry.

**Figure 24.**
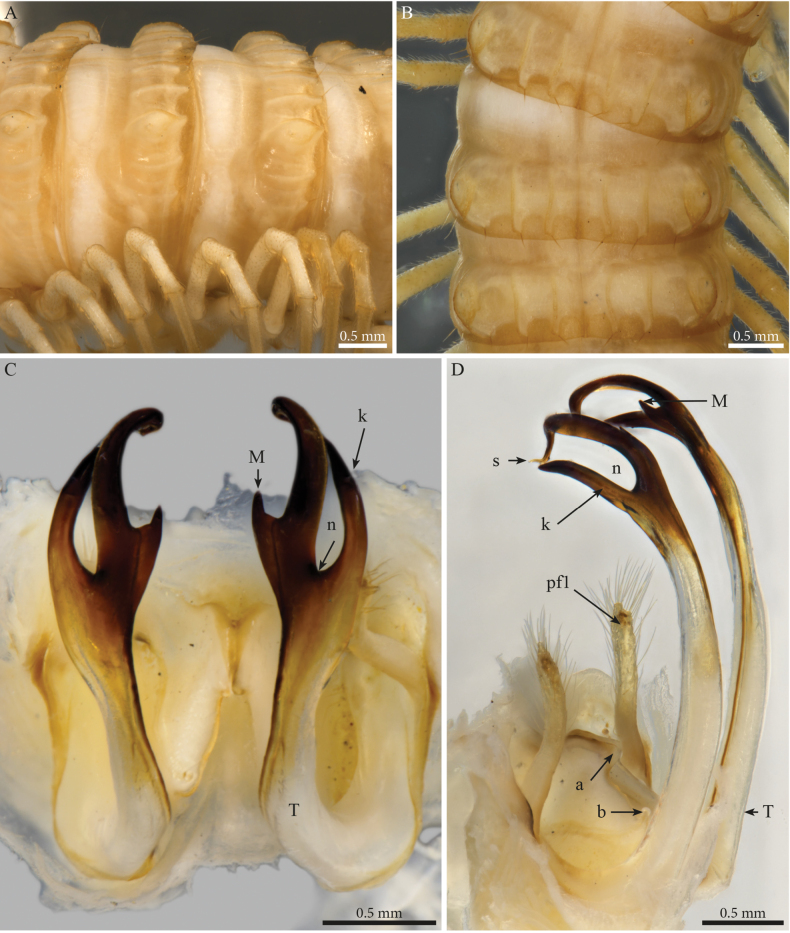
*Scotopetalumchinensis* (Stoev & Geoffroy, 2004), comb. nov., male paratype (BG-NMNHS-INV-000000006262 NMNHS) **A, B** midbody pleurotergites **A** dorsolateral view **B** dorsal view **C, D** gonopods **C** posterio-ventral view **D** posterio-lateral view. Abbreviations: a = mesal process of coxa; b = falcate mesal process of coxa; k = lateral process of the distal part of telopodite; M = mesal process of the distal part of telopodite; n = notch; pf = prefemoroidal process; s = solenomere; T = telopodite.

**Figure 25.**
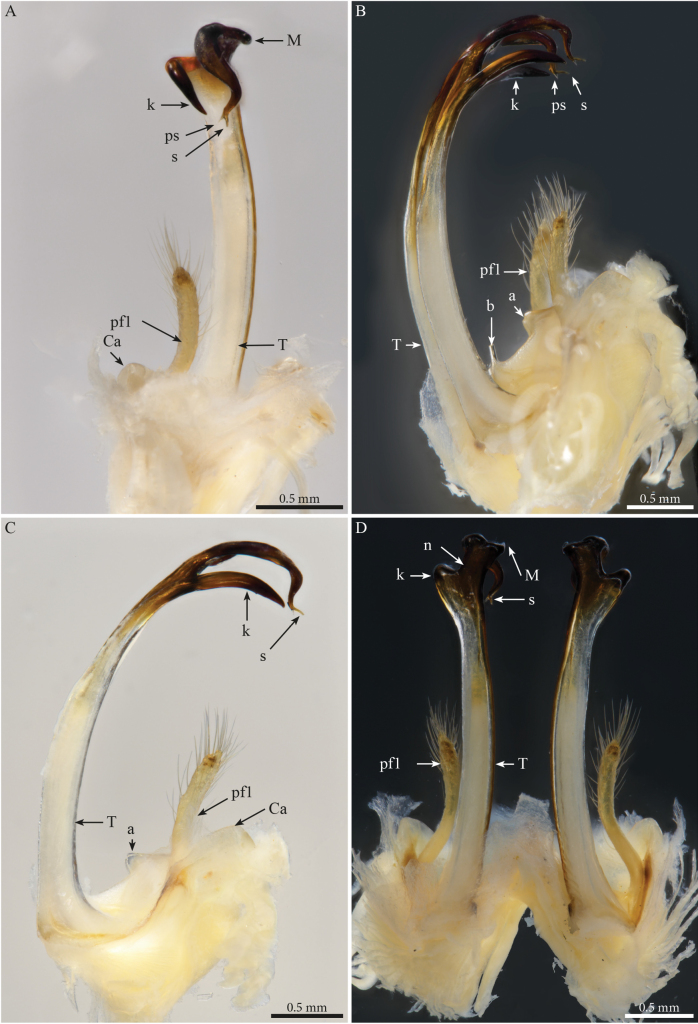
*Scotopetalumchinensis* (Stoev & Geoffroy, 2004), comb. nov., male paratype (BG-NMNHS-INV-000000006262 NMNHS) gonopods **A** left gonopod, anterior view **B** both gonopods lateral view **C** left gonopod, lateral view **D** both gonopods posterior view. Abbreviations: a =mesal process of coxa; b = falcate mesal process of coxa; Ca = anterior lobe of coxa; k = lateral process of the distal part of telopodite; M = mesal process of the distal part of telopodite; n = notch; pf = prefemoroidal process; ps = parasolenomere; s = solenomere; T = telopodite.

Figs 23 , 24 , 25 , 27G , 28

Paracortina chinensis Stoev & Geoffroy, 2004: 94, figs 1–8.

##### Studied material.

***Paratypes***: 1 male, 2 subadult females, China, Yunnan Region, Zheng Xiong County, Liao Jun Don Cave (Touristic cave), millipedes collected close to rat corpse, 17.08.1999, J. & B Lips leg. (BG-NMNHS-INV-000000006262), Stoev det. April 2004.

##### Diagnosis.

Different from *Scotopetalumwarreni* in the presence of a reduced mesal coxal process (vs absent in *S.warreni*), as well as the distal part of the telopodite. Also, in the number of ommatidia 30 ommatidia in five or six rows (vs 15 ommatidia in 6 rows), and by having 2+2 dorsal crests between poriferous crests (vs 6+6).

##### Descriptive notes.

Corresponds to the description of the species as provided by Stoev and Geoffroy (2004); see also Figs 23, 24A, B.

##### Male sexual characters.

Leg-pairs 1 and 2 reduced and more setose than the rest (Fig. 23B), leg-pair 2 with a small anterior process and posterior opening of the gonopores. Leg-pair 7 with a short mesal triangular process and a large, rounded projection on coxa, trochanter with a smaller, rounded projection covered with setae (Fig. 27G).

***Gonopods*.** Parallel (Figs 24C, D, 25). One reduced, slender, and distally uniformly setose prefemoroidal process (less than half telopodite length) (**pf1**). Coxa with a very reduced anterior lobe (**Ca**) (Fig. 25A), mesally with a triangular lobe (**a**) and a rudimentary posterior tooth (**b**) (Fig. 24D, 25B, C). Telopodite (**T**) with a uniformly slender stem, gently curved, distally with a broad notch (**n**) separating a downturned bifurcated process (Fig. 24C, D, 25) bearing the solenomere (**s**) and parasolenomere (**ps**), overpassing a shorter anteriorly directed process (**M**), and a third flattened and acuminate process (**k**) emerging laterally.

**Figure 26.**
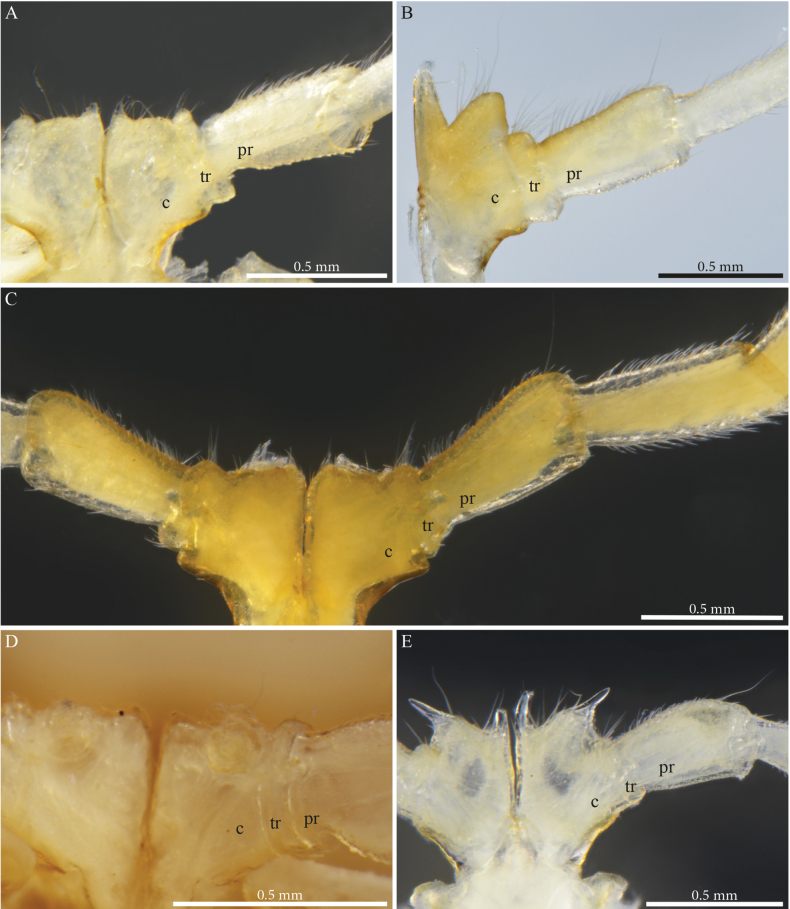
Modifications on leg-pair 6 and leg-pair 7 in adult males of species of Paracortinidae**A, B***Angulifemurunidigitis* Zhang, 1997, male **A** leg pair 6 **B** leg 7 **C***Paracortinamultisegmentata* Stoev & Geoffroy, 2004, male paratype leg pair 7 **D, E***Paracortinathallina* (Wang & Zhang, 1993), male **D** leg pair 6 **E** leg pair 7. Abbreviations: c = coxa; pr = prefemur; tr = trochanter.

**Figure 27.**
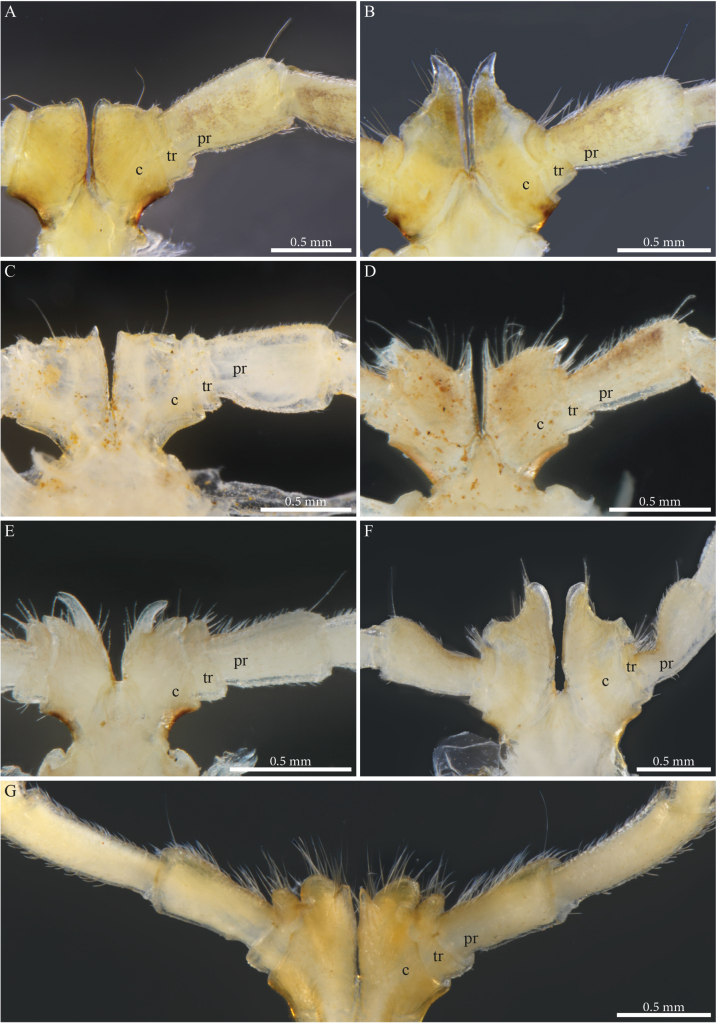
Modifications on leg-pair 6 and leg-pair 7 in adult males of species of Paracortinidae**A, B***Paracortinaviriosa* (Wang & Zhang, 1993), male **A** leg pair 6 **B** leg pair 7 **C, D***Paracortinavoluta* Wang & Zhang, 1993, male **C** leg pair 6 **D** leg pair 7 **E***Paracortinaasciformis* Akkari & Stoev, sp. nov., male holotype, leg pair 7 **F***Paracortinakabaki* Akkari & Stoev, sp. nov., male holotype, leg pair 7 **G***Scotopetalumchinensis* (Stoev & Geoffroy, 2004) comb. nov., male paratype, leg pair 7. Abbreviations: c = coxa; pr = prefemur; tr = trochanter.

##### Distribution.

Known from the caves Ke Ma Dong (Grotte du Brouillard Matinal), Liao Jun Dong (Tourist cave) and Da Hei Dong (Grande Grotte Noirre) in Zhen Xiong County, Yunnan, China.

#### ﻿ Scotopetalum warreni

Taxon classificationAnimaliaCallipodidaParacortinidae

Shear, 2000

CE4ABC56-1B71-569E-B7E4-E4FD4DFA45B5

Fig. 28

Paracortina warreni Shear, 2000: 87, fig. 1 Stoev and Geoffroy 2004: 93.

##### Diagnosis.

Different from *Scotopetalumchinensis* in the complete absence of the mesal coxal process (vs a reduced tooth-shaped process in *S.chinensis*), as well as the distal part of the telopodite. Also, the presence of 15 ommatidia in three rows and 6+6 dorsal crests between poriferous crests.

##### Descriptive notes.

Species with 53 PTs +telson, general colour yellowish-tan, head without modifications, 6+6 dorsal crests between poriferous crests, 15 ommatidia in three rows, pleurotergal setae 5+5, coxal sacs on legpairs 3–19, coxa 7 unmodified (meso-ventral thorns). Gonopods: parallel (based on Shear 2000: fig. 1). One reduced slender and distally uniformly setose prefemoroidal process (< ½ telopodite length) (**pf1**). Coxa with an anterior triangular coxal lobe (**a**). Telopodite (**T**) with a uniformly slender and gently curved stem, distally with a broad notch (**n**) separating an apical curved process pointing anteriad and a second downturned bifurcated process into solenomere (**s**) and parasolenomere (**ps**), overhanging a third flattened and acuminate process (**k**). At the level of the notch, a small hook-like process is inserted on the mesal side (Shear 2000: fig. 1, process **ttp**).

##### Comments.

The species is only known from its original description thus not much can be said about its morphology nor the configuration of the gonopods in other views besides the lateral one (Shear 2000: fig. 1). Therefore, it remains unclear if the “hook-like basal process (**ttp**)” described for this species could actually be homologues with process (**k**) of *Paracortinachinensis*.

##### Distribution.

**Figure 28.**
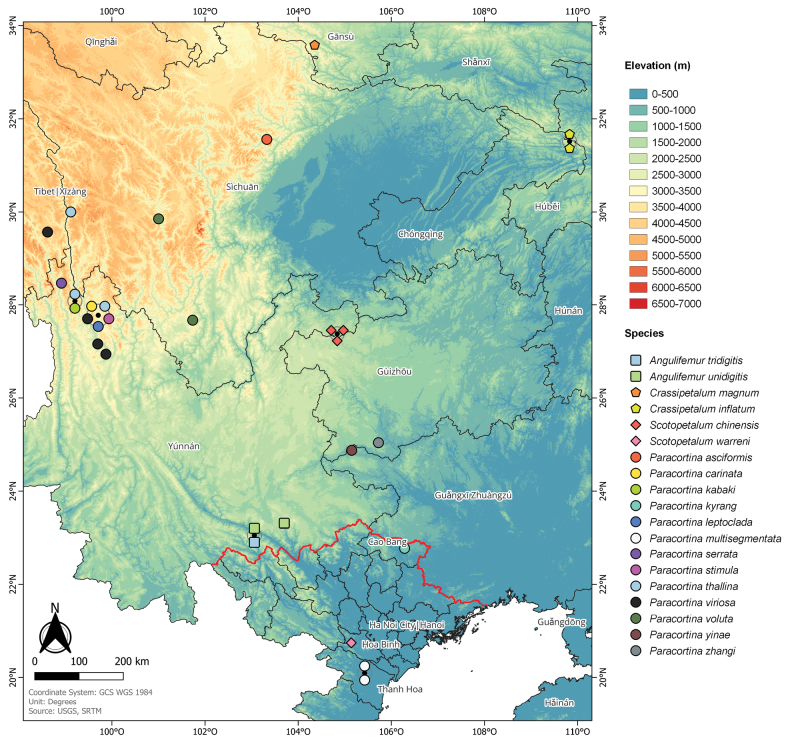
Distribution map of Paracortinidae in China and Vietnam.

Caves at Hong Mat, Hoa Binh, Vietnam. In the distribution map (Fig. 28), we used the original coordinates as stated by Shear (2000).

### ﻿Identification key to the species of the family Paracortinidae (based on gonopods)

**Table:** 

1	Telopodites proximally crossing	***Paracortinamultisegmentata***
–	Telopodites parallel, distally crossing, or diverging	**2**
2	Telopodite sinuous, broad at base, with a sharp angle at mid-length, distally twisting and narrowing *Angulifemur*	**3**
–	Telopodites parallel, sometimes distally crossing	**4**
3	Coxal process falcate, almost reaching mid-length of the telopodital stem, distal part of telopodite with two spine-like processes surrounding the solenomerite	***A.tridigitis***
–	Coxal process reduced, cone-shaped, distal part of telopodite with one well-developed tooth-shaped process	***A.unidigitis***
4	One slender prefemroidal process, coxa with an expanded rounded anterior lobe; coxal process absent; telopodite distally forked *Scotopetalum*	**5**
–	One or two prefemoroidal processes of different sizes, coxa with a long mesal process	**6**
5	Distal part of telopodite with a short process **k**; 15 ommatidia in 3 rows; 6+6 dorsal crests between poriferous crests	***S.warreni***
–	Distal part of telopodite with a longer process **k**; 30 ommatidia in 5 or 6 rows; 2+2 dorsal crests between poriferous crests	***S.chinensis***
6	Large prefemoroidal process, coxa with very large mesal process *Crassipetalum* gen. nov.	**7**
–	One or 2 significantly smaller and thinner prefemoroidal process/es, coxa with smaller lobes and a shorter falcate process	**8**
7	Two prefemoroidal processes, one large ovoid process and a smaller one; distal part of telopodite complex, with 10 apices	***C.inflatum***
–	One large and elongated prefemoroidal process, telopodite less complex	***C.magnum***
8	Telopodite with a thin stem, its distal part generally simple, curved or sinuous	**9**
–	Telopodite with a broad stem, its distal part complex, greatly expanding, with lamellae and a hook-shaped bifurcated mesal process	**10**
9	One slender prefemoroidal process, coxal process short and slender (less than half the length of the telopodite); telopodite distally gently curved, with a small, reduced lamella	***P.zhangi* and *P.yinae^1^***
–	Two slender prefemoroidal processes, coxal process larger and longer, reaching the distal part of the telopodite; telopodite sinuous, strongly narrowed towards the apex	***P.kyrang***
10	Two subequal prefemoroidal processes	**11**
–	One large and one smaller prefemoroidal processes	**12**
11	Distal part of telopodite with a broad lateral folded lamella	***P.leptoclada***
–	Distal part of the telopodite with a sinuous, differently oriented lamella	***P.thallina***
12	Distal part of the telopodite expanded proximo-distad in an axe-shaped process	***P.asciformis***
–	Distal part of the telopodite different	**13**
13	Distal part of the telopodite with a broad subrectangular or dome-shaped lobe (lateral view), of which the apical part downturned, crossing with the curved process of the solenomere	**14**
–	Distal part of the telopodite with a subquadrate/earlobe-shaped process and a broad serrated lamella	**15**
14	Distal part of the telopodite as a subrectangular plate with a horizontal apical irregular margin (lateral view)	***P.carinata***
–	Distal part of the telopodite as a dome-like process with a rounded apical margin and an anterior narrow downturned process	***P.stimula***
15	Distal part of the telopodite with two main processes	**16**
–	Distal part of the telopodite with three main processes	**17**
16	Distal part of the telopodite earlobe-shaped with a rounded lateral margin in posterior view, anterior lamella rounded and transparent, located on the lower margin part of the distal process	***P.voluta***
–	Distal part of the telopodite sub-quadrate with a slightly notched lateral margin in posterior view, anterior lamella subrectangular and darkly pigmented, located on the upper margin part of the distal process, the hook-shaped process with the solenomere longer and stouter	***P.viriosa***
17	Telopodite distal part with a subtrapezoidal serrated process and a short solenomere	***P.serrata***
–	Telopodite (T) distal part with a large subrectangular lamella with serrated margin extending anterolaterally in a double horizontal ruffle, solenomere long, slender and curved, marking an almost 180 degrees fold	***P.kabaki***

## ﻿Discussion

### ﻿On the taxonomy of the family Paracortinidae and species affinities

The Asian millipede family Paracortinidae still represents a challenge for taxonomists although it has been the subject of several treatments and reviews, including new species descriptions. Initially, this family was described to include seven species placed in three genera (Wang and Zhang 1993), viz., *Paracortina* (*P.leptoclada*, *P.voluta*), *Relictus* (*P.stimula*, *P.thallina*) and *Altum* (*P.viriosa*, *P.serrata*, *P.carinata*), all from south China. The latter two were subsequently downgraded to a subgeneric level of *Paracortina* (Wang 1996; Wang and Mauriès 1996), later formally synonymised with *Paracortina* by Stoev and Geoffroy (2004). A second genus, *Angulifemur* Zhang, 1997 containing two species, both found in caves in Yunnan, was added to the family by Zhang (1997). That article, being among the last ones of Prof. Chong-zhou Zhang and published in an obscure outlet ‘Contributions from Tianjin Natural History Museum’ remained largely unknown to the myriapodologists for nearly a decade (Stoev et al. 2014). A few years later, Shear (2000) described another monospecific callipodidan genus, *Scotopetalum*, from caves in north Vietnam which he placed in the family Schizopetalidae. Stoev (2004) doubted this placement and suggested that the genus described by Shear (2000) belonged to the family Paracortinidae. Later, Stoev and Geoffroy (2004) reviewed the family and added two new species from Vietnam and China. Here, we erect a new genus, presenting a gonopod morphology that significantly differs from the other genera of the family. viz., *Crassipetalum* gen. nov., to accommodate *C.inflatum* and the new species *Crassipetalummagnum* sp. nov.

With the present paper, the number of species in the family Paracortinidae reaches 19 in four genera, which without doubt does not reflect the real diversity of the family given how poorly studied the group is in China, Vietnam, and neighbouring countries. Perhaps what rendered the taxonomy of the family controversial as well as determining the different species and/or assigning them to genera was the fact that the original descriptions of these taxa (Wang and Zhang 1993) posed a language impediment for most taxonomists who worked on the group despite the brief species accounts given in English. The illustrations, however, proved to be useful even though one to two views were provided at most, which is often not sufficient to properly understand the morphology of the complex gonopods and most species have remained known only from their original descriptions. Here, we rediscover and document three species of the genus *Paracortina* described by Wang and Zhang (1993), viz., *P.voluta*, *P.thallina*, and *P.viriosa*; we restudy one species of the genus *Angulifemur*, *A.unidigitis* based on the type series of its junior synonym *Paracortinawangi*, and we further provide micrographs of the species *Paracortinamultisegmentata* and *Scotopetalumchinensis* comb. nov. based on their respective type materials.

In parallel to the shortcomings mentioned above, the taxonomy of this family did suffer from a choice of characters that mostly relied on variable habitual features in addition to discrepancies in the terminologies used to describe the gonopods, with no attempts to properly understand the structures and homologise them. Shear (2000) had already pointed out this issue more than twenty years ago, although he himself failed to properly understand at that time the taxonomic affinities of the genus he described, and he assigned it to the family Schizopetalidae. Wang (1996) made an isolated attempt to understand the relationships of the different species of *Paracortina*, using “cladistic analyses” but the results are not very coherent due to the poor choice of morphological characters. Here, we mostly follow the terminology proposed by Wang and Zhang (1993) and later by Stoev (2004), Stoev and Geoffroy (2004), and Enghoff et al. (2015). When possible, we tried to homologise the different structures, using the same abbreviations for those processes we think homologous or at least having a comparable placement and orientation in the gonopods. What we do not attempt at this stage is to hypothesise on the origins of the processes and we follow the already established terminology for the order. Although the gonopods in this family appear morphologically diverse, all the species assigned to Paracortinidae agree in having the following four characteristics:

1. anterior and lateral projections (lobes) of the coxa. These can be variable in shape, number but also size. Not much attention has previously been given to these structures unless for example when the anterior lobe is large and prominent;
2. coxal process, originating mesally and usually projecting mesoanteriad, that can be reduced (*Scotopetalumchinensis* comb. nov.) to very large, where it lies posterior to the telopodite (*Crassipetalummagnum* gen. nov., sp. nov.). However, in most species of the genus *Paracortina*, for example, this process is usually falcate, narrowing towards the tip and its size varies from mid-length of the telopodite to reaching its distal part;
3. one or two setose, clavate prefemoroidal process/es that appear on the anterolateral side of the gonopods in symmetrical or asymmetrical pairs or as one process, with a variable size that usually never exceeds the mid-length of the telopodite except in the genus *Crassipetalum*;
4. an elongate telopodite with a sometimes sinuous (*Angulifemur*, *Paracortinakyrang*) or curved (*Scotopetalumchinensis* comb. nov.) stem that is often expanded and complex distally (ex. *Paracortinaserrata*, *P.kabaki* sp. nov.). In all species, a median curved process detaches from the apical part and points mesoanteriad, with a bifurcation at the tip bearing the solenomere and parasolenomere. This process has a characteristic hook-shape and the left and right often intersect in the genera *Paracortina* as well as *Crassipetalum* gen. nov.

Two species of *Paracortina* are quite different from their congeners, namely *P.multisegmentata* and *P.kyrang*: both are described from Vietnam and are geographically distant from the other species found in the Chinese provinces of Guangxi, Guizhou, Sichuan, Yunnan, and Tibet Autonomous Region (Fig. 28). *Paracortinamultisegmentata* is the only representative of the family with proximally crossing telopodites and *P.kyrang* with strongly twisted telopodites. Both species are also similar in having a low coxa with rounded lobes and shorter (than most species of *Paracortina*) subfalcate processes; additionally, the shape of the coxa on leg-pair 7 has a round projection surmounted by a small spine. However, the placement of these two species in the genus *Paracortina* remains tentative until more morphological and genetic evidence is available to elucidate their taxonomic position in the family.

#### ﻿Secondary sexual characters in the family Paracortinidae

In addition to their genitalia (gonopods, vulvae), adult males and females of the family Paracortinidae present a number of secondary sexual structures that affect various parts of their external morphology (Table 2). Adult females were occasionally described in previous taxonomic treatments, and they presented enlarged pleurotergites 1 and 2, a special setal arrangement on leg-pairs 1–3 (“brushes”), with leg-pair 2 strongly reduced anterior to female genitalia or cyphopods. Prefemora of leg-pairs 3 and 4 were also described as enlarged in *P.zhangi* (Liu and Tian 2015).

**Table 2.** Secondary sexual characters in adult males of the family Paracortinidae (excluding the gonopods). modif. = modification, empty cells = no information.

Species/male sexual chars except gonopods	leg-pair 1–2	leg-pair 3	leg-pair 4	leg-pair 5	leg-pair 6	leg-pair 7	head vertex	PT6 and 7	Coxal sacs	Tarsal pads
***A.tridigitis***	“comb-like tarsal spines” (Zhang 1997)					2 processes on coxa (Zhang 1997)	no projection			
***A.unidigitis***	reduced and setose, with prefemoral and tarsal combs		a small triangular anterior process	no modif	a small triangular hyaline mesal process on coxa	a long subfalcate mesal process and a shorter larger triangular projection on coxa	no projection		3–23	tarsal pads 3–10
***C.magnum***	reduced and setose	no modif	no modif	no modif	no modif	no modif	no projection	enlarged		
***C.inflatum***	reduced and setose	no modif	no modif	no modif	no modif	no modif	no projection	enlarged		
***P.asciformis* sp. nov.**	reduced and more setose than the rest, leg-pair 2 with a small anterior process	no modif	no modif	no modif	no modif	a protruding curved mesal process pointing laterad (Fig. 18B) and a shorter subtriangular one on coxa, trochanter with an anterior triangular projection covered with setae	a large beak-shaped projection			
***P.carinata***	no modif	no modif	no modif	no modif	no modif	no modif (Wang 1996)	no projection (Wang 1996)			
***P.kabaki* sp. nov.**	reduced and more setose than the rest, leg-pair 2 with a small anterior process	no modif	no modif	no modif	no modif	a large cone-shaped and apically rounded mesal process on coxa, and an angular lateral margin separated by a notch; trochanter with an apical tuft of setae, prefemur with a strong constriction proximally on the anterior margin, then strongly swollen distally	a large beak-shaped projection			
***P.kyrang***	reduced and setose, with prefemoral and tarsal combs; leg-pair 2 with an anterior process on coxa (Nguyen et al. 2023)					a round mesal projection and a small spine	A large projection (Nguyen et al. 2023)	strongly enlarged	3–26	tarsal pads until l26
***P.leptoclada***						2 processes on coxa (Zhang 1997)	a large projection (Wang 1996)			
***P.multisegmentata***	reduced and more setose than the rest, showing prefemoral and tarsal combs	no modif	no modif	no modif	no modif	a small mesal spine on coxa and a tuft of setae on trochanter	no projection	23)	3–18	3–10
***P.serrata***						2 processes on coxa (Zhang 1997)	no projection (Wang 1996)			
***P.stimula***						no modif (Wang 1996)				
***P.thallina***	reduced and setose, showing prefemoral and tarsal combs; leg-pair 2 with an anterior process on coxa	no modif	anterior triangular process on coxa	no modif	a small mesal tooth on coxa	one mesal and one lateral slender hyaline pointed process on coxa	a triangular protruding projection on vertex			
***P.viriosa***	reduced and setose, showing prefemoral and tarsal combs; leg-pair 2 with a large anterior process on coxa	anterior triangular projection	anterior triangular projection	no modif	a small rounded mesal projection on coxa and a slight constriction of prefemur proximally on the posterior margin	strongly modified coxa, anteriorly projecting in a large mesal slightly curved horn (Fig. 27B), trochanter with strong setae	a small projection on vertex			
***P.voluta***	reduced and setose, showing prefemoral and tarsal combs; leg-pair 2 with a large anterior process on coxa	no modif	an anterior triangular projection on coxa	no modif	a short mesal triangular process and a smaller lateral process on coxa; prefemur slightly constricted proximally on the posterior margin	one pointed mesal hyaline process and a rounded projection surmounted by a similar but slightly smaller one, trochanter with a tuft of strong setae	a protruding triangular projection on vertex			
***P.yinae***	reduced and more setose than the rest, leg-pair 2 with a small anterior process				a small pointed mesal process on coxa	a slender mesal and pointed triangular process and a large rounded projection on coxa	a small beak-shaped projection on vertex		3–25	3–15
***P.zhangi***	reduced and more setose than the rest, leg-pair 2 with a small anterior process				no modif	a protruding mesal triangular process and a very large subtriangular-rounded projection on coxa	a large beak-shaped projection		3–23	3–15
***S.chinensis***	reduced and more setose than the rest, leg-pair 2 with a small anterior process	no modif	no modif	no modif	no modif	a short mesal triangular process and a large rounded projection on coxa, trochanter with a smaller rounded projection covered with setae.	no projection		3–9/10	3–18
***S.warreni***	“dense brushes of dark seate” Shear 2000					“with small mesoventral thorns” Shear 2000	no projection		3–19	3–19

Adult males present secondary sexual characters on the head vertex, pleurotergites, and legs. While males of the genera *Crassipetalum*, *Scotopetalum*, and *Angulifemur* show an unmodified head vertex, all species of the genus *Paracortina* display a distally curved triangular projection on the vertex (unknown in *P.carinata* and *P.serrata*) or a beak-shaped projection (as in *P.zhangi* and *P.yinae*). These structures are in fact not uncommon in adult male millipedes and modifications on head vertex have also been observed in the callipodidan genus *Cyphocallipus* Verhoeff, 1909 from the Pyrenees (Mauriès 1978) and a few African species of the family Trichopolydesmidae (Polydesmida) from Cameroon (Golovatch et al. 2018). A “boletiform epicranial protuberance” was described and documented for several species of the African genus *Hemisphaeroparia* (Golovatch et al. 2018, 2019) and considered diagnostic for several species of the genus (ca 15 species). This “knob-like” structure could be different sizes, placed in a depression (e.g., *H.mouanko* Golovatch, Nzoko Fiemapong, Tamesse, Mauriès & VandenSpiegel, 2018), or have a shape as a “bundle of filaments” (e.g., *H.falcata* Golovatch, Nzoko Fiemapong, Tamesse, Mauriès & VandenSpiegel, 2018). The genus *Mabocus* Chamberlin, 1951 belonging to the same family is also characterised by the presence of such a head structure (Golovatch et al. 2019). The pyrgodesmid genus *Cryptocorypha* (Polydesmida, Pyrgodesmidae) also presents modified heads in adult males (Golovatch 2019). Secondary sexual structures on the head of adult males in millipedes have also been observed in Chordeumatida, and a remarkable example is perhaps that of *Adshardicusstrasseri* Golovatch, 1981 (Antić and Makarov 2016: 20, fig. 10B, C), where the structure resembles to a certain extant those recorded in Callipodida. Other examples of Chordeumatida include the species *Bulgarosomabureschi* Verhoeff, 1926 and *B.ocellatum* (Tabacaru, 1967) (see Strasser 1962: 450, fig. 17; Tabacaru 1967).

The leg-pairs 1–7 in most cases present secondary sexual characters in adult males. In fact, leg-pairs 1 and 2 are significantly smaller in all species, and just like in the adult females they are setose, showing prefemoral and tarsal brushes. Leg-pair 2 bears the gonopores posteriorly and in some species also an anterior process on coxa (Table 2). Leg-pair 3 is in most species unmodified, except in *P.viriosa* where it presents an anterior triangular mesal projection on the coxa (Fig. 11D). In *Paracortinathallina*, *P.viriosa*, and *P.voluta*, the coxa of the same leg-pair presents an anterior mesal projection. Leg-pair 5 is unmodified in all species, while leg-pair 6 and, especially leg-pair 7, bear the most noticeable structures. When modified, leg-pair 6 displays one to two small anterior small processes on the coxa and enlarged prefemora. On the other hand, leg-pair 7 will display a more pronounced sexual dimorphism, with what appears so far to be species-specific shapes of the coxa (Table 2; Figs 26, 27). Whether these structures represent taxonomically or phylogenetically informative characters remains to be explored although their location, for instance, on the coxa of leg-pair 7 seem to be fixed for each species. It is noteworthy that the two species we considered to have an uncertain taxonomic position within the family Paracortinidae (*P.multisegmentata* and *P.kyrang*) do present more or less similar patterns of modification on coxa 7 (Fig. 26C; Nguyen et al. 2023), very different from the remaining species of *Paracortina* (typically with one or two sets of barely conspicuous to very prominent projections). Wang (1996) recorded no modifications on coxa 7 for the species *P.carinata* and *P.stimula* but this needs to be verified with the rediscovery of these species as he, in the same paper, recorded modifications in the species *P.serrata* and *P.leptoclada*, which in both cases has been revealed as erroneous (see Table 2). On the other hand, *S.chinensis* (Fig. 27G) displays a rather unique morphology of the coxa 7 that does not seem to match the description for its congener *S.warreni* (Shear 2000).

Just like the modifications of the head vertex, secondary sexual modifications on legs 1–7 in Callipodida and Chordeumatida are common and regularly appear in species descriptions. The modification of male leg-pair 7 has been considered as a good taxonomic character for the genera *Balkanopetalum* Verhoeff, 1926 (Schizopetalidae), *Dorypetalum* Verhoeff, 1900 (Dorypetalidae), *Apfelbeckia* Verhoeff, 1896 (see Stoev and Enghoff 2003, 2006, 2008), and *Heptium* Loomis, 1937 (see Loomis 1937).

The distribution of coxal sacs on leg-pairs 3–23 was proposed by Stoev and Geoffroy (2004) and Enghoff et al. (2015) as a possible character that defines the family. However, this number does not appear to be stable since different authors provided different accounts (see Table 2). In many instances, it is simply not possible to see these with certainty beyond the leg-pair 16, so whether the coxal sacs distribution represents an apomorphy for the family remains to be assessed. Many of these structures appear in the Callipodida’s sister group Chordeumatida but they have never been the subject of a close morphological or anatomical study; thus any interpretation of their putative function remains tentative though it is almost certain that they play a role during mating and copulation as they are completely absent in juveniles and immature specimens. The coxal sacs are known to serve for sperm storage in Chordeumatida (Koch 2015).

### ﻿Troglomorphic species and convergent characters

Nine species of the family Paracortinidae were described from caves, including all members of the genera *Crassipetalum*, *Scotopetalum*, and *Angulifemur*, and four species of the genus *Paracortina* (*P.multisegmentata*, *P.kyrang*, *P.yinae*, and *P.zhangi*). These species exhibit (to a different extent in each species) classical troglomorphic characters, such as depigmentation of the cuticle, reduction in the number of ommatidia, elongation of legs and antennae, and, in extreme cases, even an increase in the number of pleurotergites, reaching 85 in *P.multisegmentata*. The highest degree of troglomorphism is observed in *Angulifemurunidigitis*, *S.warreni*, and especially *P.kyrang*. Completely blind species are, however, lacking, also the case of Sinocallipodidae, the most speciose family in the area (Stoev and Enghoff 2011).

### ﻿Geographical distribution

The family Paracortinidae is the most diverse family of the order Callipodida in Southeast Asia. The highest species diversity was found in the Chinese provinces of Yunnan (3 genera, 10 species) and Sichuan (1 genus, 3 species). Representatives of the family are also found in the provinces of Gansu, Guzhou, and Guangxi, as well as in the Tibet Autonomous Region. Outside China, the family is also found in North Vietnam in Thanh Hóa and Cao Bang provinces. The northern distribution limit of the family runs through the middle of Sichuan, ca the 32^nd^ North parallel, the only exception being *Crassipetalummagnum* sp. nov., with an even more northerly locality in the Zhou-qu area of Gansu Province (Fig. 28). It is possible that the family reaches further northeast, as female callipodidans from Cisian-shan, 25 km south of Nanjing (Jiangsu Province) were reported as *Bollmania* sp. by Golovatch (1981), 12 years before the description of the family Paracortinidae. This find was later commented upon by Shear et al. (2003) as likely belonging to an undescribed genus and species of the family Paracortinidae (see also Stoev and Enghoff 2005). In the south, the family reaches ~ 20 N parallel in the province Thanh Hóa in Vietnam, where the species *S.warreni* and *P.multisegmentata* have been reported. From west to east, the family extends from eastern Tibet and western parts of Sichuan and Yunnan to northeastern Vietnam, with the easternmost locality being *Crassipetaluminflatum* from Chongqing in China. Thus, until now, members of the family are known from ~ 97^th^ to 106^th^ Eastern meridian. If the Nanjing area record turns out to be truly representative of the family, it will be the easternmost of all. It is likely that the distribution of the family Paracortinidae also cover northern Myanmar and eastern Laos, and possibly northern Sichuan Province, central and western Yunnan, northwestern Guizhou, and Guangxi. In Vietnam, the family is probably also distributed in the northwestern parts, in the provinces of Hà Giang, Lao Cai, for example.

## Supplementary Material

XML Treatment for Paracortinidae

XML Treatment for Angulifemur

XML Treatment for Angulifemur tridigitis

XML Treatment for Angulifemur unidigitis

XML Treatment for Crassipetalum

XML Treatment for Crassipetalum magnum

XML Treatment for Crassipetalum inflatum

XML Treatment for Paracortina

XML Treatment for Paracortina asciformis

XML Treatment for Paracortina carinata

XML Treatment for Paracortina kabaki

XML Treatment for Paracortina kyrang

XML Treatment for Paracortina leptoclada

XML Treatment for Paracortina multisegmentata

XML Treatment for Paracortina serrata

XML Treatment for Paracortina stimula

XML Treatment for Paracortina thallina

XML Treatment for Paracortina viriosa

XML Treatment for Paracortina voluta

XML Treatment for Paracortina yinae

XML Treatment for Paracortina zhangi

XML Treatment for Scotopetalum

XML Treatment for Scotopetalum chinensis

XML Treatment for Scotopetalum warreni

## Footnotes

Species separated by the absence vs presence of a spiniform coxal process on leg-pair 6 and the processes on coxa 7 in adult males (Liu and Tian 2015)

## ﻿Additional information

### Author ORCIDs

Nesrine Akkari https://orcid.org/0000-0001-5019-4833

Oliver Macek https://orcid.org/0000-0002-8146-5373

Pavel Stoev https://orcid.org/0000-0002-5702-5677

### Data availability

All of the data that support the findings of this study are available in the main text.
